# Mapping the metabolomic and lipidomic changes in the bleomycin model of pulmonary fibrosis in young and aged mice

**DOI:** 10.1242/dmm.049105

**Published:** 2022-01-25

**Authors:** Jelena Weckerle, Sergio Picart-Armada, Stephan Klee, Tom Bretschneider, Andreas H. Luippold, Wolfgang Rist, Christian Haslinger, Holger Schlüter, Matthew J. Thomas, Bartlomiej Krawczyk, Francesc Fernandez-Albert, Marc Kästle, Daniel Veyel

**Affiliations:** 1 Boehringer Ingelheim Pharma GmbH & Co. KG, Department Immunology and Respiratory Disease research, Birkendorfer Straße 65, 88397 Biberach an der Riß, Germany; 2 Boehringer Ingelheim Pharma GmbH & Co. KG, Global Computational Biology and Digital Sciences, Birkendorfer Straße 65, 88397 Biberach an der Riß, Germany; 3 Boehringer Ingelheim Pharma GmbH & Co. KG, Department Drug Discovery Sciences, Birkendorfer Straße 65, 88397 Biberach an der Riß, Germany

**Keywords:** Pulmonary fibrosis, Bleomycin, Metabolomics, Lipidomics, Aging

## Abstract

Alterations in metabolic pathways were recently recognized as potential underlying drivers of idiopathic pulmonary fibrosis (IPF), translating into novel therapeutic targets. However, knowledge of metabolic and lipid regulation in fibrotic lungs is limited. To comprehensively characterize metabolic perturbations in the bleomycin mouse model of IPF, we analyzed the metabolome and lipidome by mass spectrometry. We identified increased tissue turnover and repair, evident by enhanced breakdown of proteins, nucleic acids and lipids and extracellular matrix turnover. Energy production was upregulated, including glycolysis, the tricarboxylic acid cycle, glutaminolysis, lactate production and fatty acid oxidation. Higher eicosanoid synthesis indicated inflammatory processes. Because the risk of IPF increases with age, we investigated how age influences metabolomic and lipidomic changes in the bleomycin-induced pulmonary fibrosis model. Surprisingly, except for cytidine, we did not detect any significantly differential metabolites or lipids between old and young bleomycin-treated lungs. Together, we identified metabolomic and lipidomic changes in fibrosis that reflect higher energy demand, proliferation, tissue remodeling, collagen deposition and inflammation, which might serve to improve diagnostic and therapeutic options for fibrotic lung diseases in the future.

## Abstract (precis)

**Editor's choice:** Using bleomycin-induced lung injury as a mouse model for idiopathic pulmonary fibrosis, this study identifies metabolomic and lipidomic changes in fibrosis reflecting higher energy demand, proliferation, tissue remodeling, collagen deposition and inflammation.

## INTRODUCTION

Idiopathic pulmonary fibrosis (IPF) is a chronic and progressive fibrotic lung disease that leads to irreversible stiffening of the interstitial tissue, impaired gas exchange and, ultimately, respiratory failure. The main characteristics of IPF include fibroblast foci accumulation, aberrant extracellular matrix (ECM) deposition and alveolar epithelial cell damage (Barratt et al., 2018; Lederer and Martinez, 2018). In IPF patients not receiving anti-fibrotic therapy or lung transplantation, the median survival is 3.5-4.5 years following diagnosis (Lancaster et al., 2019; Nathan et al., 2011; Perez et al., 2010). The two currently approved therapeutics, pirfenidone and nintedanib, slow down the progression of the disease and extend the median survival of patients to 8.5 years (Fisher et al., 2017; Lancaster et al., 2019). However, there is no available drug that can reverse the fibrosis and repair the existing lung injury. Extensive research is performed to unravel the currently not well-understood pathogenic and molecular mechanisms causing IPF.

The most frequently used animal model of IPF is bleomycin-induced lung injury and fibrosis in mice, although it does not fully recapitulate the disease pathology (Moore et al., 2013). Bleomycin, a chemotherapeutic drug, leads to epithelial cell death in the first 3 days after administration, followed by excessive inflammatory infiltrates in days 3-9 and, ultimately, to fibroblast activation, ECM deposition and fibrosis with a peak around days 14-21 after injury (Tashiro et al., 2017). Most studies to date focus on the bleomycin-treated young mice (2-3 months of age), but little is known about the fibrotic response in aged animals. Few studies suggested that old mice upon bleomycin instillation develop more severe lung fibrosis, measured by increased collagen deposition, inflammation and mortality, compared to young mice (Redente et al., 2011; Stout-Delgado et al., 2016). Older age represents one of the risk factors for IPF in humans, and the median age of patients at diagnosis is 66 years (Ley et al., 2011). Moreover, aging at the cellular level including telomere shortening, increased endoplasmic reticulum stress, mitochondrial dysfunction and reactive oxidative species accumulation in alveolar epithelial cells also contributes to disease development (Armanios et al., 2007; Kinnula et al., 2005; Korfei et al., 2008; Patel et al., 2015).

An increasing number of studies suggest that the dysfunction of metabolic pathways contributes to the pathogenesis of chronic lung diseases. In addition, lipids play crucial roles in the lung, as structural components, energy storage, surfactant molecules and signaling mediators. Alteration of lipid content has been implicated in pathophysiological processes (Agudelo et al., 2020). Previous metabolomic studies in human IPF lungs identified reduced glutathione metabolism, reflecting oxidative stress (Kang et al., 2016), and increased collagen-building amino acids and arginine metabolism, indicating ECM remodeling (Kang et al., 2016; Zhao et al., 2017). In addition, metabolic shift from glycolysis to lactate production (Kang et al., 2016; Zhao et al., 2017), accumulated fatty acids, downregulated β-oxidation and decreased tricarboxylic acid (TCA) cycle indicated impaired energy pathways in IPF lungs (Zhao et al., 2017). Reduced sphingolipid and ceramide metabolism suggested gross structural remodeling of fibrotic lungs (Zhao et al., 2017). Similarly, metabolomic profiling of bleomycin-treated mouse lungs identified increases in collagen synthesis and protein degradation (Milara et al., 2015; Seo et al., 2019), as well as increased energy production (Aichler et al., 2018; Seo et al., 2019; Sun et al., 2018). In addition, oxidative stress and glutathione metabolism were shown to be increased (Milara et al., 2015; Seo et al., 2019). Although the inflammatory response was mostly upregulated upon bleomycin challenge, one study found a decrease in prostaglandins (Milara et al., 2015; Sun et al., 2018). Lipid perturbations in bleomycin-treated lungs, including increased fatty acids, phosphatidylcholine (PC) and phosphatidylethanolamine (PE), indicated membrane turnover and proliferation (Milara et al., 2015).

Detailed characterization of deregulated metabolites and lipids in lung fibrosis ultimately improves understanding of pathogenic disease mechanisms, which could lead to faster diagnosis and the discovery of novel therapeutic targets. To comprehensively dissect treatment- and age-related differences in metabolic and lipid pathways in lung fibrosis, we performed global metabolomic and lipidomic analysis of whole lungs of young (3 months of age) and old (21 months) bleomycin-treated and control mice. Interestingly, we observed minimal contribution of aging to the metabolic and lipidomic profile of the fibrotic changes 21 days after bleomycin treatment. However, we identified bleomycin-induced metabolic changes involved in lung injury and tissue remodeling in both young and old animals that reflect cellular and pathological features of IPF. These included increased biomacromolecule degradation and collagen turnover, glycolytic shift to lactate production, increased β-oxidation, perturbed lipid metabolism and increased inflammatory eicosanoids. In addition, we compared in detail our bleomycin-induced lung fibrosis data to published human IPF metabolomic data (Kang et al., 2016). We identified metabolites previously not mentioned in the context of IPF that could play important roles in the development of lung fibrosis. Thus, this study offers a detailed and comprehensive understanding of the bleomycin model for studying IPF pathology and treatment options, extending its scope to metabolic processes, and advises on the correct use of age in preclinical studies to explore novel metabolomic targets.

## RESULTS

### Bleomycin treatment induces perturbation of major metabolic pathways and lipid classes in the lung

**Fig. 1.**
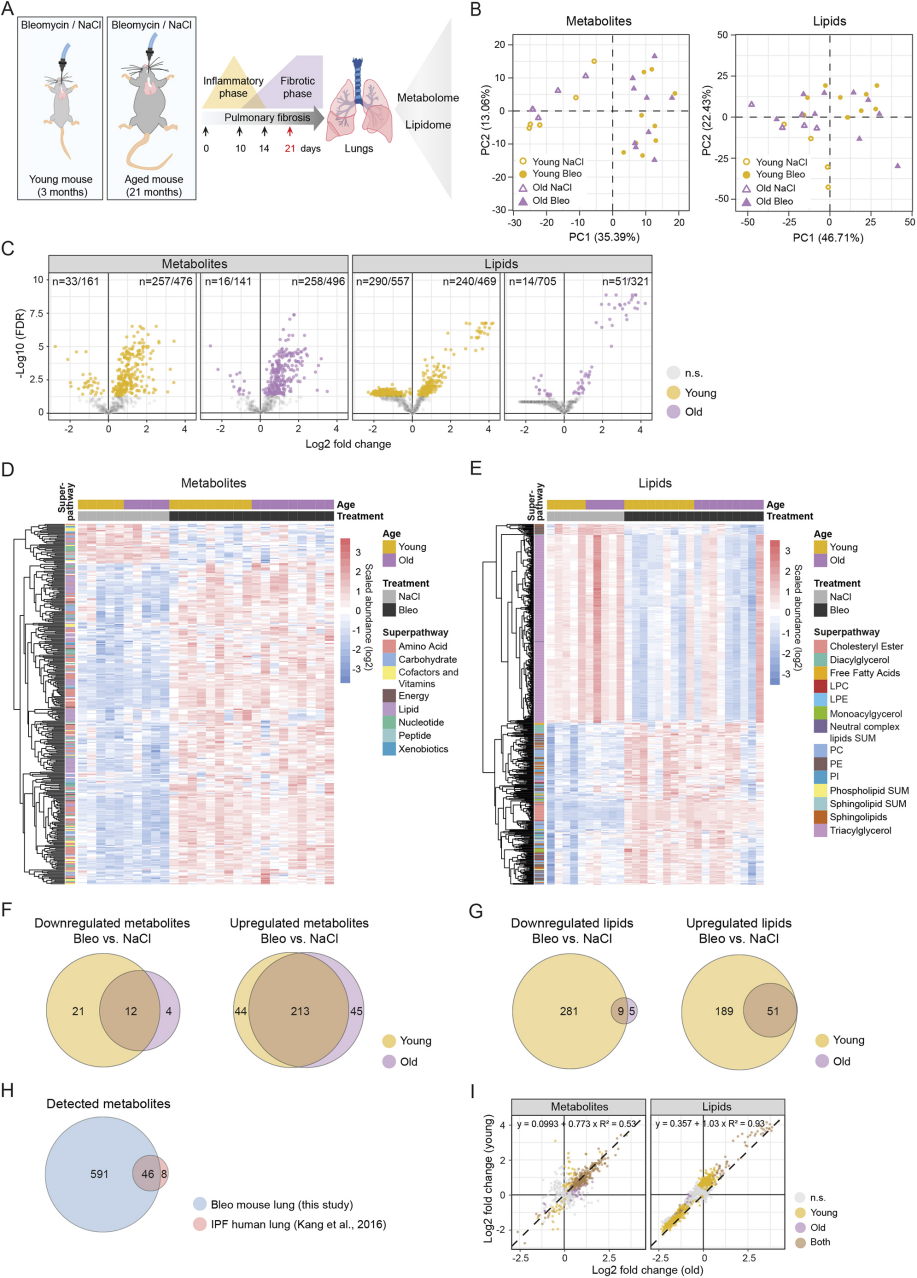
**Detected and significantly deregulated metabolites and lipids in the lungs of young and old bleomycin-treated mice.** (A) Schematic illustration of the study design. Young and old mice were instilled with bleomycin or saline solution (NaCl control), and the whole lungs were isolated on day 21 after treatment in the peak of the fibrotic phase. Metabolomic and lipidomic analyses were performed by mass spectrometry. (B) Principal component (PC) analysis of all lung samples, indicating separation between controls and bleomycin-treated animals, but not by age. (C) Volcano plots of all detected metabolites and lipids in young and old bleomycin-treated lungs. Significant entities are colored (one-way ANOVA), and the numbers for upregulated and downregulated metabolites and lipids are indicated. (D,E) Heat maps showing log2 scaled abundance of significantly deregulated metabolites (D) and lipids (E) in bleomycin-treated compared to control lungs in any age group. (F,G) Venn diagrams of significantly downregulated and upregulated metabolites (F) and lipids (G) of bleomycin-treated compared to control lungs in young and old mice. The metabolites and lipids that are significantly deregulated only in young, only in old or in both age groups are listed in Table S4. (H) Venn diagram of total detected metabolite numbers in this study in bleomycin-treated mouse lungs and in the published idiopathic pulmonary fibrosis (IPF) study on human lungs compared to controls (Kang et al., 2016). (I) Scatter plots showing log2 fold changes of detected metabolites and lipids in young and old mice with their best linear fit (regression line, equation and coefficient of determination). FDR<0.05 in C-I. Bleo, bleomycin; FDR, false discovery rate; LPC, lysophosphatidylcholine; LPE, lysophosphatidylethanolamine; Neutral complex lipids SUM, total cholesteryl ester, monoacylglycerol, diacylglycerol, triacylglycerol, free fatty acid; n.s., not significant; PC, phosphatidylcholine; PE, phosphatidylethanolamine; Phospholipid SUM, total phosphatidylcholine, lysophosphatidylcholine, phosphatidylethanolamine, lysophosphatidylethanoamine, phosphatidylinositol; PI, phosphatidylinositol; Sphingolipid SUM, total sphingomyelin, ceramide, dihydroceramide, lactosylceramide, hexosylceramide.

To comprehensively study metabolomic and lipidomic changes in pulmonary fibrosis in the context of aging, we instilled young and old mice intratracheally with 0.7 mg/kg and 0.63 mg/kg bleomycin sulphate in saline solution (NaCl), respectively (Fig. 1A). The dose of bleomycin for old mice was adjusted to achieve similar fibrotic phenotype as that in young mice. This was demonstrated by the comparable expression of fibrotic markers *Col1a1* and *Il6* (Fig. S1A) and lung function parameters [resistance and forced expiratory volume (FEV), 0.1] (Fig. S1B) in young and old mice. Control mice received saline solution only (Fig. 1A). We used perfused, cryo-powdered whole lungs at day 21 after bleomycin treatment to analyze levels of 637 metabolites and 1026 lipid species by mass spectrometry (Tables S1 and S2). Principal component analysis (PCA) shows that the samples were well separated by the treatment, but not by the age of the animals in both metabolomic and lipidomic datasets (Fig. 1B). We performed one-way analysis of variance (ANOVA) in each age group [false discovery rate (FDR)<0.05] and detected 339 metabolites and 595 lipids significantly deregulated in bleomycin-treated lungs compared to controls (Fig. 1C; Fig. S2A,B, Table S3). Heat maps of all significant metabolites indicated that most metabolites were upregulated upon bleomycin treatment (Fig. 1D; Fig. S2B), while lipids were up- and downregulated (Fig. 1E; Fig. S2B). Among the significantly deregulated metabolites in bleomycin-treated compared to control samples, there was a large overlap of those detected in young and old mice (odds ratio 20.9, *P*<2.2×10^−16^, two-sided Fisher's exact test). However, some metabolites were only deregulated in young or only in old animals (Fig. 1F,G; Table S4). Metabolites that were significantly downregulated only in young mice included creatine phosphate, *S*-methylcysteine, carnitine, phosphoenolpyruvate, 3-phosphoglycerate, nicotinamide adenine dinucleotide (NAD+) and flavin adenine dinucleotide (FAD) (Table S4). Galactose 6-phosphate and 4-hydroxyglutamate were specifically downregulated in old mice (Table S4). Metabolites that were only upregulated in young mice included heme, arachidonate, glucose, maltose, creatinine and choline (Table S4). In old mice, we found increased fructose 6-phosphate, mannose 6-phosphate, glucose 6-phosphate, 13-hydroxyoctadecadienoic acid (13-HODE), 9-hydroxyoctadecadienoic acid (9-HODE), gluconate, xanthosine, β-alanine, cysteine-glutathione disulfide and other metabolites (Table S4). From the metabolomic analysis, the highest numbers of deregulated metabolites in bleomycin-treated compared to control lungs were found among amino acid, lipid, nucleotide and carbohydrate superpathways (Fig. S2C).

Surprisingly, the number of significantly deregulated lipid entities (FDR<0.05) found in young bleomycin-treated mice (*n*=530) compared to controls was much higher than that in old mice (*n*=65) (Fig. 1C,G; Fig. S2A). This striking effect is mainly driven by lower *P*-values in young mice, causing the FDR values to be below our chosen cut-off of 5% (Fig. S2A,D, Table S3). Those differences probably arise from higher heterogeneity of bleomycin response in old animals, and from certain lipid classes, including triacylglycerols (TAGs), being highly intercorrelated, therefore affecting the statistical analysis and the FDR calculation. Nevertheless, the overlap between the deregulated lipids detected in bleomycin-treated old and young mice was significant (odds ratio 12.5, *P*=4.3×10^−13^, two-sided Fisher's exact test). We identified TAGs as a downregulated lipid class only in young bleomycin-treated lungs (Table S4). We did not detect any significantly upregulated lipids that were specific to old mice (Fig. 1G; Table S4). In contrast, in young mice, upregulated lipids included phosphatidylinositol species (PIs), PEs, diacylglycerol species (DAGs) and, interestingly, dipalmitoylphosphatidylcholine [DPPC; PC(16:0/16:0)], the major phospholipid of the lung surfactant (Table S4). Highest numbers of differential lipids between bleomycin-treated and control mice were identified within TAG, PC, PE, cholesteryl ester (CE) and sphingolipid classes (Fig. S2D).

Out of 637 metabolites detected in bleomycin-treated lungs and controls in this study, 46 overlapped with metabolites detected in IPF lungs compared to controls in a previous study (Kang et al., 2016) (Fig. 1H; Fig. S3A,B). Of note, the study on IPF lungs identified only 54 metabolites in total, and eight of those were not detected in our study (Fig. 1H). Nevertheless, the significant changes and their fold change directions were similar between the bleomycin model and IPF lungs (Fig. S3A,B). Out of 20 metabolites that were significant in both studies, 18 had the same direction of change, while two were deregulated in opposite directions (Fig. S3B), supporting the translatability of the bleomycin model for clinical research.

Notably, we only detected cytidine as a differentially downregulated metabolite and no significantly deregulated lipids when comparing bleomycin-treated old and young lungs (Fig. S3C,D, Table S5). The highly similar response to bleomycin treatment in old and young mice was also evident when comparing the fold changes in both age groups (Fig. 1I), showing a Pearson's correlation of 0.73 and 0.96 for metabolites and lipids, respectively (*P*<2.2×10^−16^ for both). Overall, the peak fibrotic phase at day 21 of the disease age does not play a major role in the regulation of metabolic pathways.

### Pathway analysis reveals bleomycin treatment- and age-specific changes

**Fig. 2.**
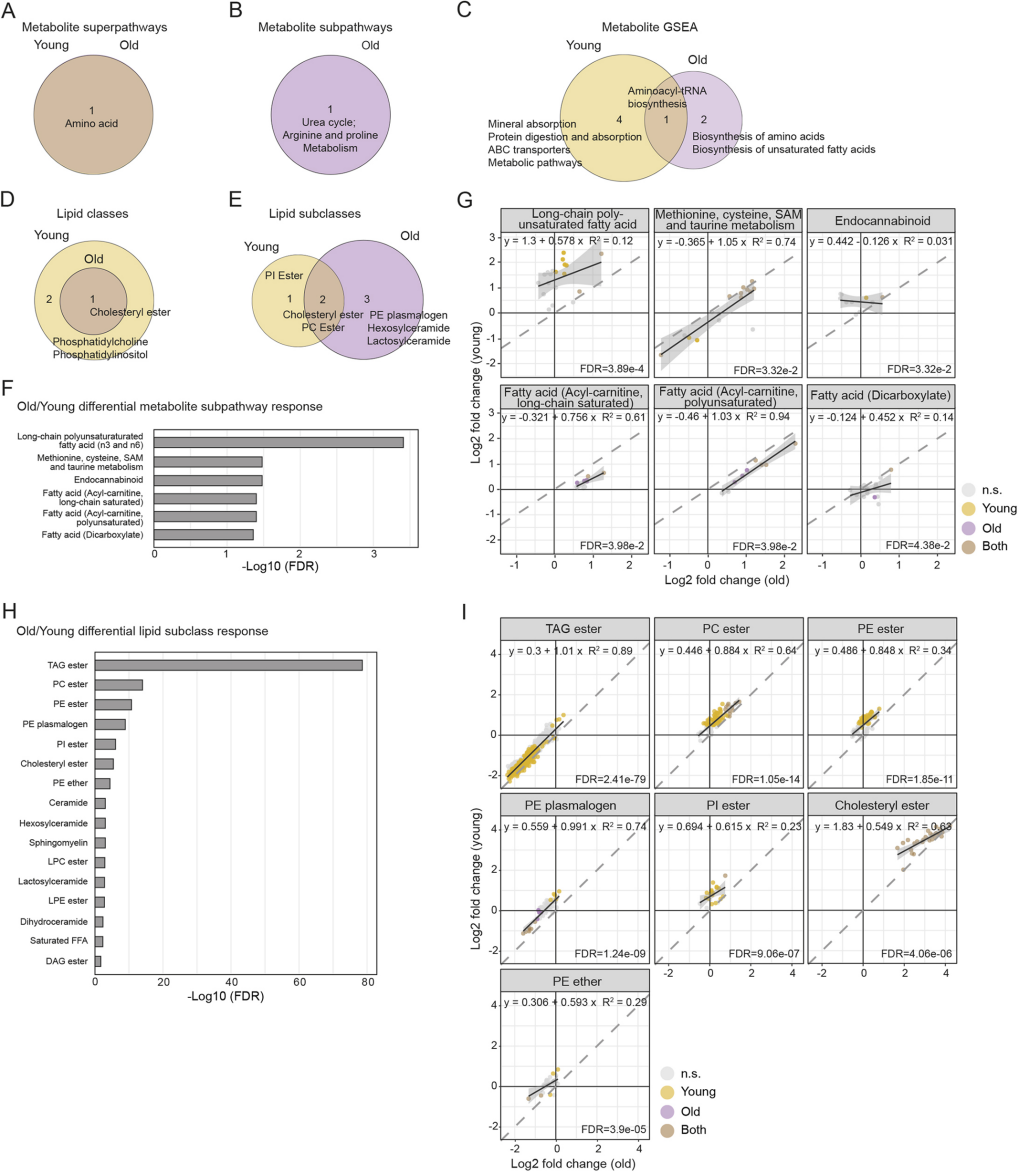
**Over-representation of metabolic pathways and lipid classes upon bleomycin treatment and differential pathway responses in young and old mice.** (A,B) Over-representation analysis of metabolic superpathways (A) and subpathways (B) in bleomycin-treated mice compared to controls. (C) GSEA of metabolic pathways in bleomycin-induced lungs compared to controls using KEGG annotations. (D,E) Over-representation analysis of lipid classes (D) and subclasses (E) in bleomycin-treated mice compared to controls. (F,H) Differential metabolic subpathway (F) and lipid subclass (H) responses among age groups according to fold change distributions in young and old mice. (G,I) Scatter plots showing log2 fold changes of detected metabolic subpathways (G) and lipid subclasses (I) in young and old mice, with their best linear fit (regression line, equation and coefficient of determination). The FDR values indicate differential pathway responses. The results for A-F and H are provided in Table S6. *q*<0.05 in A-E; FDR<0.05 in F-I. DAG, diacylglycerol; FDR, false discovery rate; GSEA, gene set enrichment analysis; n.s., not significant; PC, phosphatidylcholine; PE, phosphatidylethanolamine; PI, phosphatidylinositol; SAM, *S*-adenosylmethionine; TAG, triacylglycerol.

To identify metabolic pathways and lipid classes that were affected in fibrotic lungs compared to controls, we performed pathway over-representation analysis using custom annotations for metabolites termed super- and subpathways (Table S1) and classes and subclasses for the lipids (Table S2). For metabolites, we found significant over-representation of the amino acid pathway in both age groups (Fig. 2A; Table S6). In old mice specifically, the subpathway urea cycle, arginine and proline metabolism was over-represented (Fig. 2B; Table S6). In addition, applying gene set enrichment analysis (GSEA) using Kyoto Encyclopedia of Genes and Genomes (KEGG) annotations to metabolomics data revealed enrichment of aminoacyl-tRNA biosynthesis in both age groups (Fig. 2C; Table S6). In young mice, mineral absorption, protein digestion and absorption, ABC transporters and metabolic pathways were also enriched. Interestingly, in old mice, enriched pathways included biosynthesis of amino acids and biosynthesis of unsaturated fatty acids (Fig. 2C; Table S6). Over-represented lipid classes in both old and young bleomycin-treated mice included CE, while PC and PI were only significantly enriched in young animals (Fig. 2D; Table S6). Particularly, subclasses PE plasmalogen, hexosylceramide and lactosylceramide were enriched in old bleomycin-treated mice, PI ester only in young mice, and CE and PC ester in both age groups (Fig. 2E; Table S6).

Next, to detect any age-related differences in pathway perturbations between young and old bleomycin-treated mice, we compared the distributions of fold changes of all pathway entities (Table S6). We found most significant differential distribution of fold changes between old and young mice in the fatty acid metabolism and methionine, cysteine, *S*-adenosylmethionine (SAM) and taurine metabolic pathways (Fig. 2F,G). Among lipid classes, the strongest fold change differences in old and young lungs were identified in TAG ester, PC ester, PE ester, PE plasminogen, PI ester, CEs and others (Fig. 2H,I).

To give context to the deregulated metabolites, we performed a network-based analysis termed FELLA (Picart-Armada et al., 2018). FELLA has built a knowledge graph from the KEGG database that connected 10,974 entities: 4103 metabolites, 5592 reactions, 1003 enzymes, 184 KEGG modules and 92 metabolic pathways. Metabolites found deregulated in both young and old mice were mapped to the network. FELLA extracted a proximal subnetwork, based on statistically adjusted network propagation. The subnetwork consisted of two large, separated components (Fig. S4). The first one contained amino acid metabolism intermediates, urea cycle and polyamines, TCA cycle and vitamin B6 metabolism components (Fig. S4). The second network included carbohydrates metabolism, phospholipids, glycosphingolipids and aminosugars, as well as many nucleotides that were enriched using this method (Fig. S4).

Together, these results indicated that bleomycin treatment induced many changes to metabolomic and lipidomic pathways. Although the fibrotic response at the metabolic level was comparable between old and young animals, some pathways were only altered in old or only in young mice, or with different intensity fold changes between the age groups.

### Metabolomic and lipidomic changes reflect increased biomacromolecule turnover and tissue remodeling in fibrosis

**Fig. 3.**
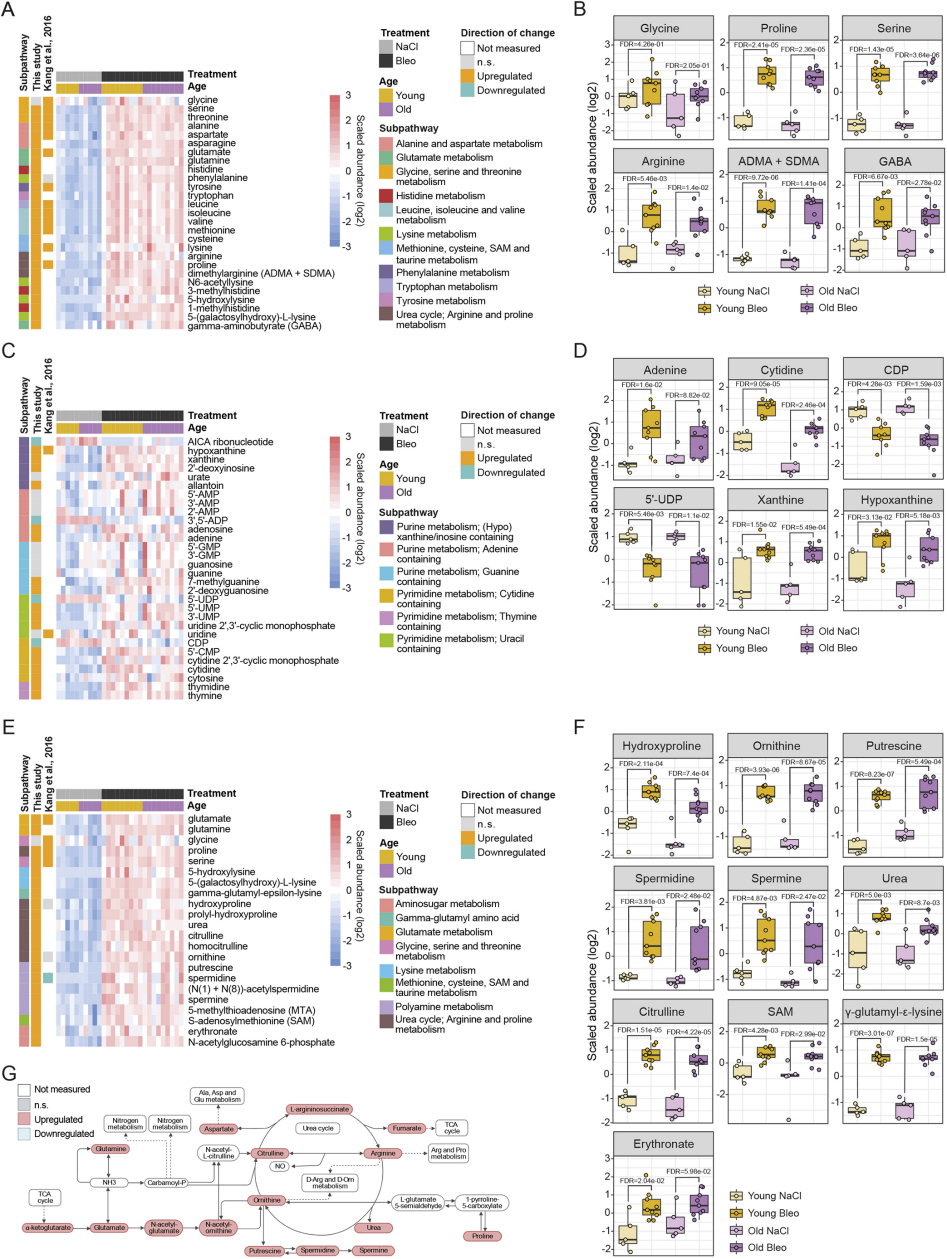
**Breakdown of macromolecules and degradation of collagen and extracellular matrix (ECM) reflect tissue turnover and repair in bleomycin-treated lungs.** (A,B) Heat map (A) and box plots (B) showing log2 scaled abundance of indicated metabolites involved in protein degradation in bleomycin-treated and control lungs. (C,D) Heat map (C) and box plots (D) showing log2 scaled abundance of indicated metabolites involved in nucleic acid turnover in bleomycin-treated and control lungs. (E,F) Heat map (E) and box plots (F) showing log2 scaled abundance of indicated metabolites involved in collagen and ECM turnover in bleomycin-treated and control lungs. (G) Simplified scheme of arginine metabolism and urea cycle with connections to other metabolic pathways. In B, D and F, the lower and higher hinges represent the first and third quartiles, respectively; the median is indicated by the intermediate bar. The whiskers extend up to 1.5 times the interquartile range from each hinge; more distant data points are displayed as outliers. FDR<0.05. ADMA, asymmetric dimethylarginine; ADP, adenosine diphosphate; AICA, 5-aminoimidazole-4-carboxamide; Ala, alanine; AMP, adenosine monophosphate; Arg, arginine; Asp, aspartate; Bleo, bleomycin; CDP, cytidine diphosphate; CMP, cytidine monophosphate; FDR, false discovery rate; GABA, gamma-aminobutyrate; Glu, glutamate; GMP, guanosine monophosphate; n.s., not significant; Orn, ornithine; Pro, proline; SAM, *S*-adenosylmethionine; SDMA, symmetric dimethylarginine; TCA, tricarboxylic acid; UDP, uridine diphosphate; UMP, uridine monophosphate.

In bleomycin-treated lungs compared to control lungs, we detected changes in metabolites and lipids that reflect a tissue turnover and repair signature. Building blocks for biomacromolecules and pathways supporting their synthesis and degradation were collectively increased. For example, we detected changes indicating increased protein turnover. Strikingly, we found all proteinogenic amino acids, except glycine, significantly upregulated compared to controls (Fig. 3A,B). Moreover, several upregulated metabolites indicated protein degradation, such as asymmetric (ADMA) and symmetric (SDMA) dimethylarginine, 1-methylhistidine, 3-methylhistidine and N6-acetyllysine (Fig. 3A,B).

Further, we found nucleic acid metabolism disturbed in bleomycin-injured lungs compared to control lungs. We detected an increase in adenine, cytidine and thymine, and also their nucleosides adenosine, cytosine and thymidine (Fig. 3C,D). In addition, uridine monophosphate (UMP) and cytidine monophosphate (CMP) were upregulated in bleomycin-treated lungs compared to control lungs, whereas the respective diphosphates were downregulated (Fig. 3C,D).

Interestingly, in addition to the turnover of cellular biomacromolecules, we observed an increase in ECM degradation products and enhanced metabolic activity involved in collagen turnover in bleomycin-induced lung fibrosis compared to controls. We found increased modified lysine [5-(galactosylhydroxy)-L-lysine and 5-hydroxylysine], hydroxyproline, prolyl-hydroxyproline and γ-glutamyl-ε-lysine, a product of transglutaminase enzyme (TG2) that crosslinks proteins, making them resistant to proteolysis (Fig. 3E,F). An increase in hyaluronic acid degradation products from the ECM, erythronate and *N*-acetylglucosamine 6-phosphate (Fig. 3E,F) also indicated increased ECM remodeling.

Further, we observed enhanced metabolic activity supporting collagen synthesis. The most abundant amino acids in collagen are glycine and proline, building the repeating motif Gly-Pro-X, where X can be any other amino acid (Shoulders and Raines, 2009). Proline on certain positions is hydroxylated into hydroxyproline, which enables formation of the triple helix and renders increased thermal stability of collagen (Xu et al., 2019). We found glycine to be the sole amino acid not significantly increased upon bleomycin treatment compared to controls (Fig. 3A,B,E). Moreover, 3-phosphoglycerate, a biosynthetic precursor of glycine from glycolysis, was depleted (Fig. 5A,C), indicating metabolic flux from glycolysis to glycine metabolism, supporting collagen synthesis. Furthermore, proline synthesis from arginine metabolism and the urea cycle were also strongly increased (Fig. 3E-G), supporting collagen synthesis.

**Fig. 4.**
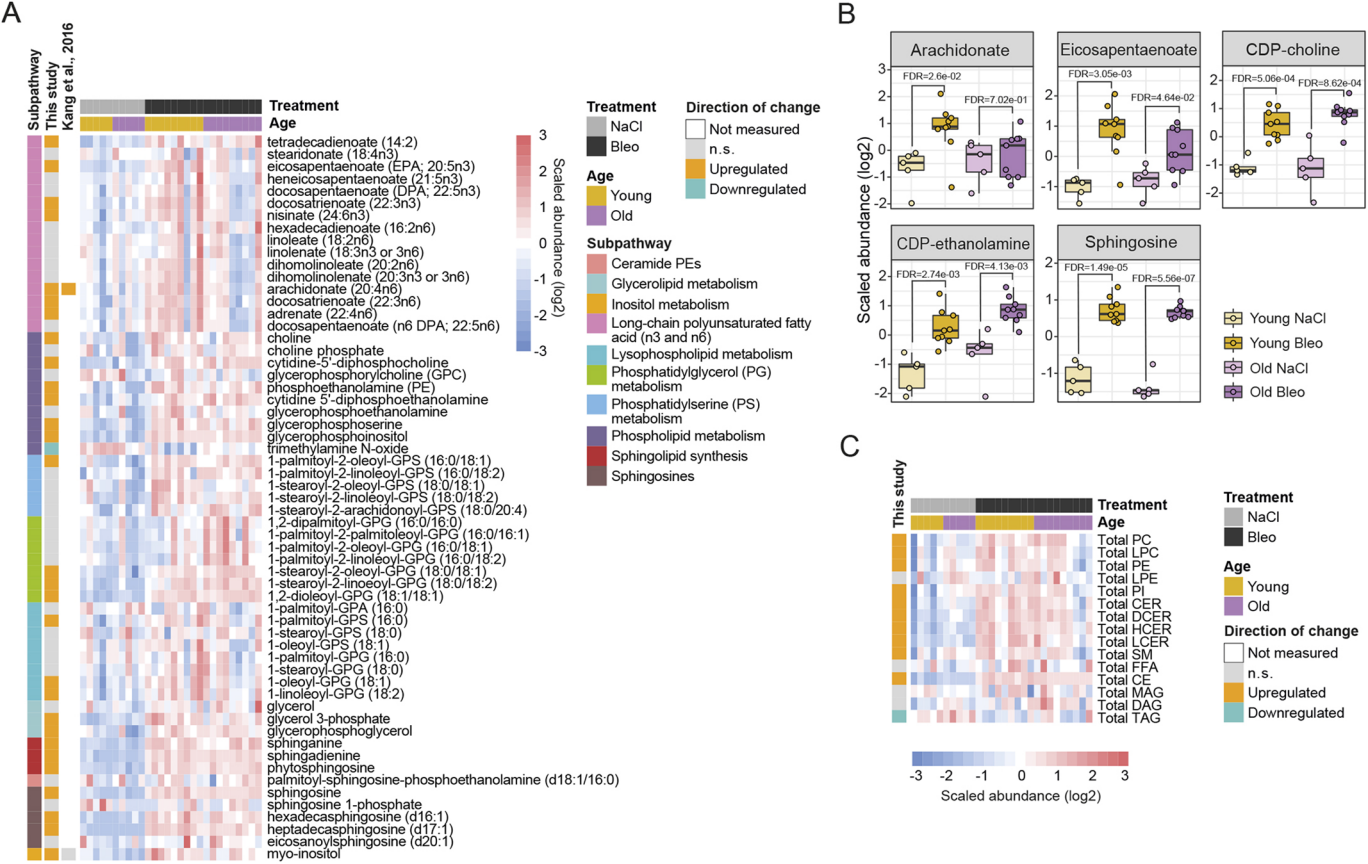
**Tissue turnover and repair lipid signature in bleomycin-treated lungs.** (A,B) Heat map (A) and box plots (B) showing log2 scaled abundance of indicated lipids involved in tissue turnover and remodeling in bleomycin-treated and control lungs. In B, the lower and higher hinges represent the first and third quartiles, respectively; the median is indicated by the intermediate bar. The whiskers extend up to 1.5 times the interquartile range from each hinge; more distant data points are displayed as outliers. (C) Heat map showing log2 scaled abundance of total lipid classes in bleomycin-treated and control lungs. FDR<0.05. Up- and downregulated metabolites in heat maps are annotated according to the changes in young mice (fold change and FDR<0.05). Bleo, bleomycin; CDP, cytidine diphosphate; CE, cholesteryl ester; CER, ceramide; DAG, diacylglycerol; DCER, dihydroceramide; FDR, false discovery rate; FFA, free fatty acid; GPA, glycerophosphate; GPC, glycerophosphocholine; GPG, glycerophosphoglycerol; GPS, glycerophosphoserine; HCER, hexosylceramide; LCER, lactosylceramide; LPC, lysophosphatidylcholine; LPE, lysophosphatidylethanolamine; MAG, monoacylglycerol; n.s., not significant; PC, phosphatidylcholine; PE, phosphatidylethanolamine; PI, phosphatidylinositol; SM, sphingomyelin; TAG, triacylglycerol.

**Fig. 5.**
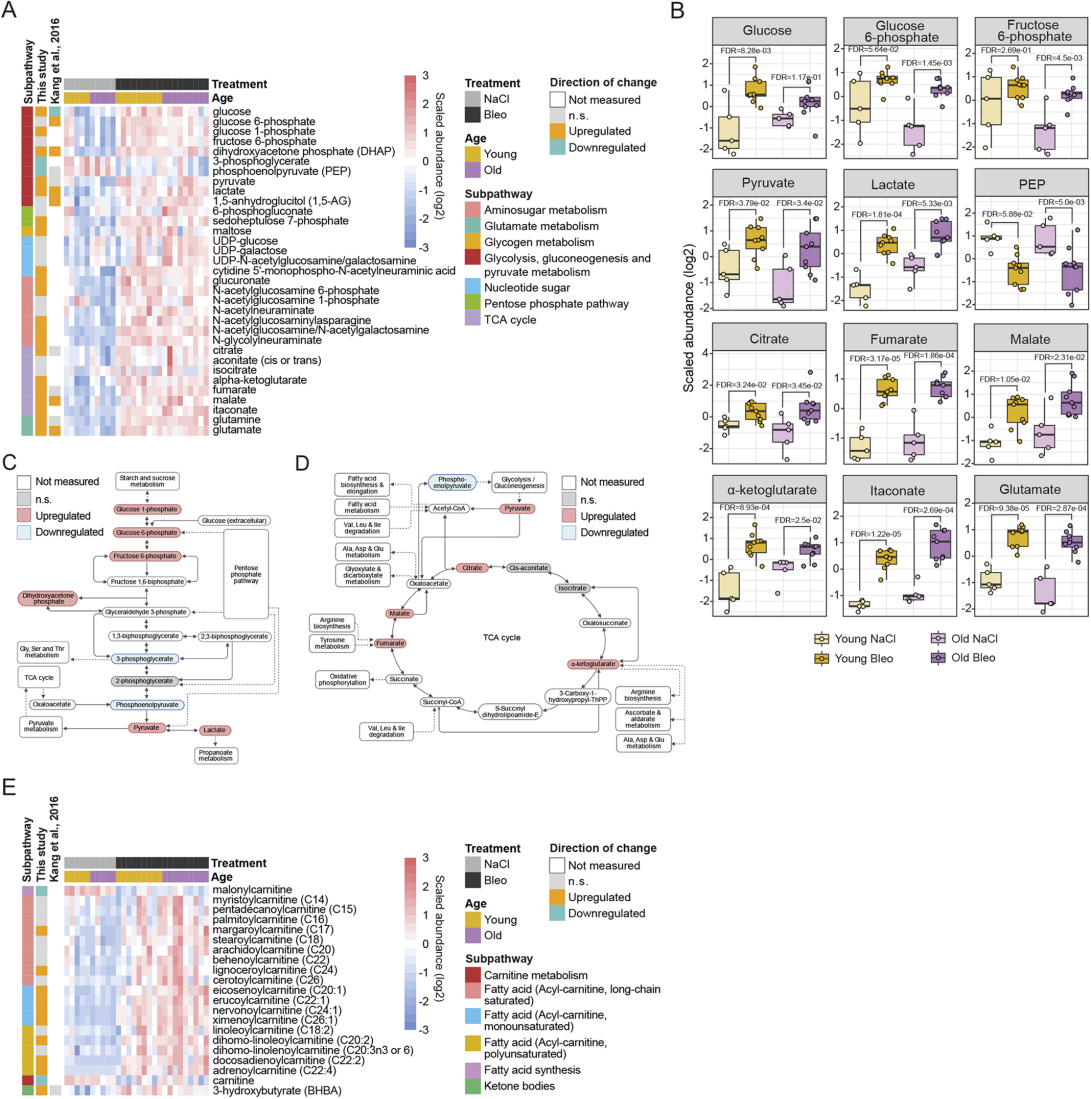
**Metabolomic reprogramming of energy pathways in bleomycin-treated lungs.** (A,B) Heat map (A) and box plots (B) showing log2 scaled abundance of indicated metabolites involved in energy-producing pathways in bleomycin-treated and control lungs. In B, the lower and higher hinges represent the first and third quartiles, respectively; the median is indicated by the intermediate bar. The whiskers extend up to 1.5 times the interquartile range from each hinge; more distant data points are displayed as outliers. (C,D) Simplified scheme of the glycolysis pathway (C) and TCA cycle (D) and the connections to other metabolomic pathways. (E) Heat map showing log2 scaled abundance of indicated fatty acid β-oxidation metabolites in bleomycin-treated and control lungs. Up- and downregulated metabolites in heat maps are annotated according to the changes in young mice (fold change and FDR<0.05). FDR<0.05. Ala, alanine; Asp, aspartate; Bleo, bleomycin; FDR, false discovery rate; Glu, glutamate; Gly, glycine; Ile, isoleucine; Leu, leucine; n.s., not significant; PEP, phosphoenolpyruvate; Ser, serine; TCA, tricarboxylic acid; Thr, threonine; UDP, uridine diphosphate; Val, valine.

We found several changes indicating synthesis and breakdown of membrane and storage lipid molecules. Increased lipid breakdown was reflected in higher levels of polyunsaturated fatty acids, particularly tetradecaienoate, eicosapentaenoate, docosatrienoate and arachidonate (Fig. 4A,B) and a prominent decrease in TAGs (Fig. 4C). In addition, upregulation of lysophospholipids [palmitoyl-glycerophosphoserine (GPS), oleoyl-glycerophosphoglycerol (GPG), linoleoyl-GPG] and several glycerophosphodiesters (glycerophosphoserine, glycerophosphoinositol, glycerophosphoethanolamine, glycerophosphorylcholine) indicated degradation of all major phospholipid classes. Higher levels of several sphingosine species may have resulted from sphingolipid degradation (Fig. 4A-C). Increased intermediates of the phospholipid synthesis pathway [glycerol-3-phosphate, phosphoethanolamine, choline phosphate, cytidine diphosphate (CDP)-choline and CDP-ethanolamine] and sphinganine indicated enhanced phospholipid and sphingolipid lipid synthesis, respectively (Fig. 4A,B). However, malonylcarnitine as a surrogate marker for malonyl-CoA was decreased (Fig. 5E). This suggested that enhanced tissue remodeling is supported by the breakdown of complex lipids. Analysis of the total lipid class abundances revealed increase within classes of CEs, hexosylceramides and PIs, whereas TAGs were strongly decreased (Fig. 4C). In addition, total PCs, PEs and ceramides were significantly increased in young mice (Fig. 4C). However, total lipid content did not change after bleomycin treatment in either age group (Fig. S5).

In summary, we identified a strong turnover and tissue remodeling signature in all classes of biomacromolecules in the lungs after bleomycin-induced lung fibrosis. Interestingly, we also detected a metabolic signature indicative of increased ECM turnover in fibrotic lungs.

### Bleomycin treatment induces a metabolic shift supporting increased energy demand

Metabolic reprogramming is well described in fibrotic tissues, similar to cancer cell metabolism (Zhao et al., 2018). Remodeling and growing tissues compensate the increasing energy demand by undergoing a metabolic switch to aerobic glycolysis, upregulation of TCA cycle and oxidative phosphorylation. We detected an increase in several glycolysis intermediates (glucose 6-phosphate, fructose 6-phosphate, dihydroxyacetone phosphate, pyruvate and lactate) in bleomycin-treated lungs compared to control lungs (Fig. 5A-C), indicating a metabolic shift from glycolysis to pyruvate metabolism and lactate production (Fig. 5B). We found increased levels of glutamine and glutamate, suggesting that increased glutaminolysis upregulated α-ketoglutarate to support energy metabolism via the TCA cycle (Fig. 5A,B,D). Moreover, a strong increase in itaconate conversion from *cis*-aconitate indicated enhanced TCA cycle metabolite production (Fig. 5B). In addition, elevated levels of sedoheptulose 7-phosphate and 6-phosphogluconate, metabolites of the pentose phosphate pathway and several aminosugar metabolites (*N*-acetylglucosamine/*N*-acetylgalactosamine, *N*-acetlyglucosamine 6-phosphate and *N*-acetylglucosamine 1-phosphate) reflected upregulation of cellular energy-producing pathways in the fibrotic lung (Fig. 5A).

Increased catabolism of fatty acids via β-oxidation provides acetyl-CoA for the TCA cycle and leads to increased energy production. Many acyl-carnitines were upregulated in bleomycin-treated lungs compared to control lungs, while free carnitine levels were decreased and 3-hydroxybutyrate (BHBA) levels were elevated, reflecting increased β-oxidation (Fig. 5E).

In summary, increase in glycolysis, TCA cycle, pentose phosphate pathway and aminosugar metabolites, together with the accumulation of acyl-carnitines and reduced levels of TAGs, reflects upregulated energy production to meet the increased energy demand of the fibrotic lungs.

### Enhanced inflammatory and immune modulatory molecule production in fibrotic lungs

**Fig. 6.**
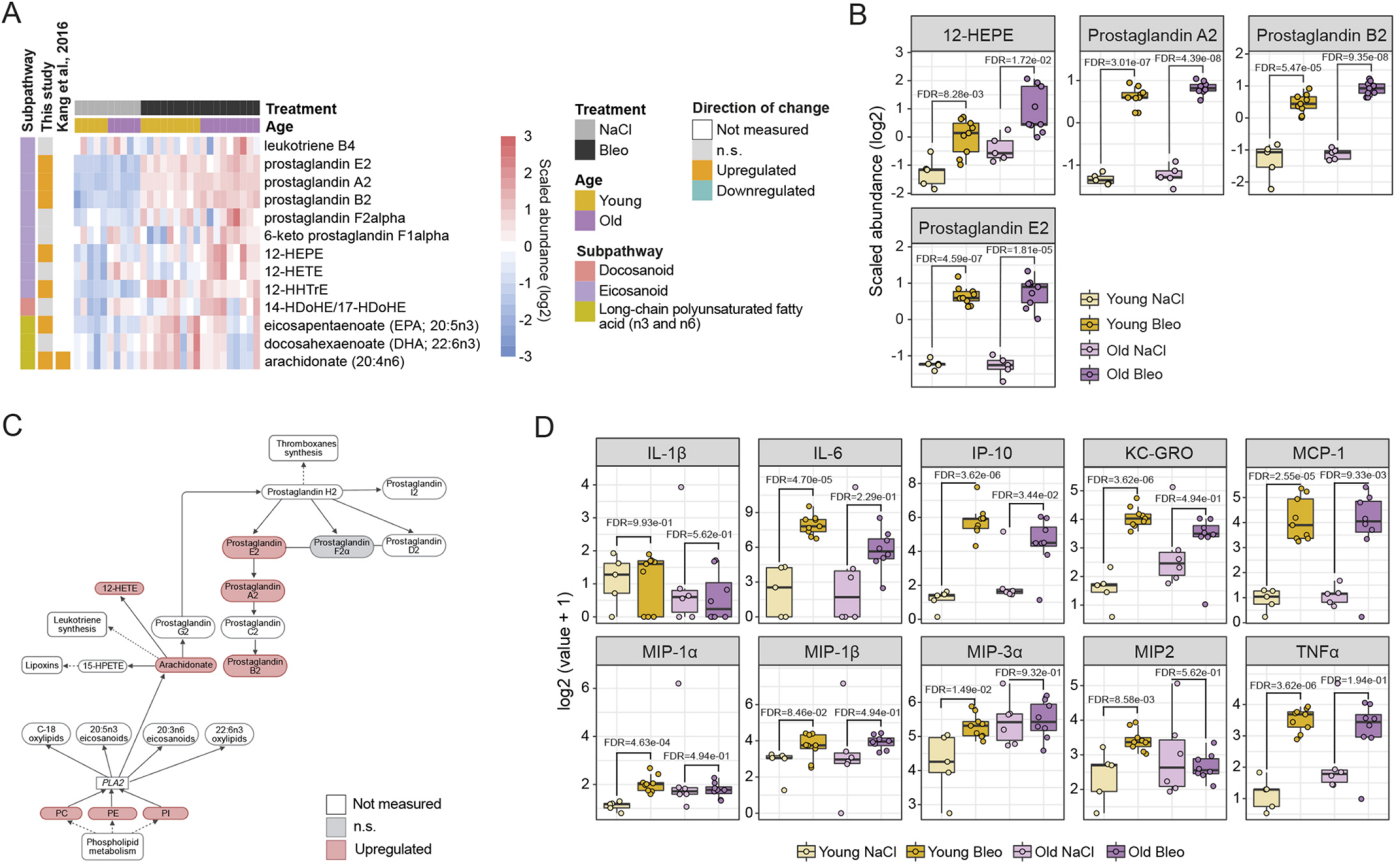
**Altered inflammation and immune modulation pathways in fibrotic lungs.** (A,B) Heat map (A) and box plots (B) showing log2 scaled abundance of indicated eicosanoid lipids involved in inflammation and immune modulation in bleomycin-treated and control lungs. In B, the lower and higher hinges represent the first and third quartiles, respectively; the median is indicated by the intermediate bar. The whiskers extend up to 1.5 times the interquartile range from each hinge; more distant data points are displayed as outliers. FDR<0.05. (C) Simplified scheme of the eicosanoid pathway with the connections to other metabolomic pathways. (D) Box plots showing log2 values+1 of indicated cytokines/chemokines in the bronchioalveolar lavage (BAL) of young and old animals upon bleomycin treatment on day 21. Each dot represents an individual animal: Old Bleo, *n*=8; Old NaCl, *n*=6; Young Bleo, *n*=9; Young NaCl, *n*=5. Significance was assessed with a one-way ANOVA, stratified by age, and *P*-values were corrected using the FDR. Up- and downregulated metabolites in heat maps are annotated according to the changes in young mice (fold change and FDR<0.05). Bleo, bleomycin; FDR, false discovery rate; n.s., not significant; PC, phosphatidylcholine; PE, phosphatidylethanolamine; PI, phosphatidylinositol; PLA2, phospholipase A2; 12-HEPE, 12-hydroxyeicosapentaenoic acid; 12-HETE, 12-hydroxyeicosatetraenoic acid; 12-HHTrE, 12-hydroxyheptadecatrienoic acid; 14-HDoHE/17-HDoHE, 14-hydroxydocosahexaenoic acid/17-hydroxydocosahexaenoic acid; 15-HPETE, 15-hydroperoxyarachidonic acid.

Eicosanoids are signaling molecules derived by oxidation of arachidonic acid (AA) or other 20 carbon unit-long polyunsaturated fatty acids like dihomo-γ-linolenic acid and eicosapentaenoic acid (EPA) (Dennis and Norris, 2015). This group of lipids was traditionally seen as a pro-inflammatory component of the innate immune response, but further studies have elucidated some eicosanoids and docosanoids with anti-inflammatory functions (Lawrence et al., 2002). We found EPA and AA, as well as intermediates of eicosanoid synthesis, 12-hydroxyeicosapentaenoic acid (12-HEPE) and 12-hydroxyheptadecatrienoic acid (12-HHTrE), significantly increased in bleomycin-treated lungs compared to control lungs (Fig. 6A-C). Prostaglandin E2, A2 and B2 were also significantly upregulated in both young and old mice upon bleomycin treatment (Fig. 6B), indicating increased immune modulatory signaling via this class of lipids, potentially enhancing the inflammatory response in the fibrotic lungs. To verify the inflammation status according to the increased inflammatory lipids, we measured the ten most relevant cytokines and chemokines in lung inflammation in the bronchioalveolar lavage (BAL) of the respective animals. We detected increased levels of the pro-inflammatory cytokines interleukin 6 (IL-6), interferon γ-induced protein 10 (IP-10; also known as CXCL10) and tumor necrosis factor α (TNFα; also known as TNF), as well as the chemokines keratinocyte chemoattractant (KC)/human growth-regulated oncogene (GRO; also known as CXCL1) and monocyte chemoattractant protein-1 (MCP-1; also known as CCL2) in bleomycin-treated animals in comparison to the controls. IL-1β, as well as the chemokines macrophage inflammatory protein 1α (MIP-1α; also known as CCL3), MIP-1β (also known as CCL4), MIP-3α (also known as CCL20) and MIP2 (also known as CXCL2) were not significantly different between the treatment groups (Fig. 6D).

## DISCUSSION

Here, we provide a detailed characterization of the metabolomic and lipidomic response of young and aged mouse lungs, 21 days after exposure to bleomycin. We profiled 637 metabolites and 1026 lipids by mass spectrometry, representing the most comprehensive study of metabolome and lipidome changes in this pulmonary fibrosis model to date. Interestingly, we found more than half of the metabolites and lipids (339 and 595, respectively) significantly deregulated in bleomycin-treated lungs compared to control lungs in both age groups (FDR<0.05), suggesting a strong impact of bleomycin-induced fibrosis on lung metabolism.

Observed perturbations of metabolomic and lipidomic pathways after bleomycin treatment correspond to reported changes in IPF patient lungs, supporting the use of this model for IPF research. We detected a metabolic signature reflective of tissue turnover and repair, and, interestingly, also collagen and ECM remodeling. Many proteinogenic amino acids were increased, indicating higher protein degradation and collagen synthesis. In a previously published metabolic study on IPF lungs, glycine, serine, glutamate and proline, along with other proteinogenic amino acids, were increased (Kang et al., 2016). Excessive production of ECM proteins and collagen has previously been linked to increased *de novo* serine and glycine synthesis (Hamanaka et al., 2018; Nigdelioglu et al., 2016). Inhibition of the glycine synthesis enzyme phosphoglycerate dehydrogenase (PHGDH) attenuated features of pulmonary fibrosis (Hamanaka et al., 2018; Nigdelioglu et al., 2016), indicating the crucial role of this pathway in fibrogenesis. Serine and glycine are synthesized from glucose through oxidation of 3-phosphoglycerate (Hamanaka et al., 2018; Rabinowitz and Mutlu, 2019). We found decreased levels of 3-phosphoglycerate in glycolysis and increased levels of serine, indicating that higher amino acid synthesis rate reduces the levels of this intermediate metabolite. In addition, glutamine conversion to glutamate and further into proline was required for production of collagen in transforming growth factor β (TGFβ; also known as TGFB1)-treated fibroblasts (Hamanaka et al., 2019). Of note, both glutamate and glutamine were significantly increased in our analysis of bleomycin-induced lung injury, similar to published IPF lung data (Kang et al., 2016; Zhao et al., 2017). Arginine in the urea cycle serves as the precursor for the synthesis of polyamines (spermidine and spermine) and proline for collagen synthesis (Endo et al., 2003). In line with the study by Zhao and co-workers (Zhao et al., 2017), we detected upregulation of arginine metabolism and the urea cycle intermediates, including putrescine, spermidine and spermine. This also reflects metabolic mechanisms to counteract oxidative stress and inflammation (Baek et al., 2020). However, in another metabolic study on IPF lungs, spermidine levels were decreased compared to those in healthy controls (Kang et al., 2016).

Collagen breakdown intermediates γ-glutamyl-ε-lysine, 5-hydroxylysine, 5-(galactosylhydroxy)-L-lysine, hydroxyproline and prolyl-hydroxyproline were increased in bleomycin-treated lungs compared to control lungs. Increase in hydroxyproline and prolyl-hydroxyproline in the IPF lungs was previously detected at a metabolomic level (Zhao et al., 2017). This indicates not only increased collagen turnover, but also suggests structural remodeling and crosslinking of collagen fibers. It was shown that formation of γ-glutamyl-ε-lysine isopeptide bonds leads to protein crosslinking, resistance to proteolysis and increased stiffness of the ECM (Bergamini et al., 2011; Fell et al., 2021; Wang and Griffin, 2012). Inhibition of TG2, an enzyme that performs crosslinking and prevents the release of TGFβ1 from its latent form in the ECM, reduced ECM deposition and myofibroblast activation (Fell et al., 2021).

Besides molecules with known implication in the pathogenesis of IPF, we also detected upregulation of other metabolites that require further confirmation from patient data. For example, erythronate, together with other products of hyaluronic acid degradation, was increased in the context of pulmonary hypertension in rats (Rafikova et al., 2016). Therefore, the detected increase in hyaluronic acid degradation products in our study also supports increased tissue and ECM remodeling in a bleomycin model of IPF.

Interestingly, we detected an increase in γ-aminobutyric acid (GABA) in bleomycin-induced lung injury compared to control lungs. Pulmonary neuroendocrine cells were considered the only source of GABA in the upper airways in primates (Barrios et al., 2019; Hewitt and Lloyd, 2021), where it stimulates mucus secretion (Sui et al., 2018). Expression of GABA-synthetizing enzyme was detected in AT2 cells, suggesting that epithelial cells represent a source in the distal lung (Jin et al., 2006). GABA treatment of rat lungs after ventilator-induced lung injury decreased pulmonary edema, improved alveolar fluid clearance, provided protection against alveolar epithelium damage and decreased apoptosis (Chintagari and Liu, 2012). Thus, the GABA increase in bleomycin-treated lungs suggests a counter-reaction to dampen bleomycin-related epithelial damage. However, little is known about the role of GABA in IPF, and further studies are needed to unveil its functions and potential therapeutic effect for pulmonary fibrosis.

We found alterations in nucleotide metabolism, indicating increased DNA damage and apoptosis and activation of repair mechanisms. Enrichment of nucleotide metabolism in bleomycin-treated lungs was also confirmed by the FELLA analysis. However, those changes were not reported in previous studies on IPF lungs and could represent the effect of bleomycin on DNA damage. Thus, this needs further confirmation in IPF patients. Interestingly, we detected upregulation of xanthine and hypoxanthine, which were associated with wound healing in intestinal epithelial cells (Fernandez et al., 2018). This could indicate the initiation of the lung repair processes to reduce the bleomycin-induced epithelial damage. Targeted inhibition of xanthine-converting enzyme xanthine oxidoreductase (XOR; also known as XDH) accelerated wound healing in diabetic wound models (Weinstein et al., 2015). Because IPF is often considered as an impaired wound healing process (Betensley et al., 2016; Chambers, 2008), it would be interesting to investigate the role of the xanthine pathway in this process.

Alterations in lipid metabolism and fatty acids in the alveolar epithelium induce oxidative stress, inflammation and pulmonary fibrosis (Whitsett et al., 2010). In our lipidomic analysis, the upregulation of fatty acid acyl-carnitines and decrease in TAGs indicated increased lipid degradation and enhanced β-oxidation upon bleomycin treatment underlying the increased energy demand of fibrotic lungs. Similarly, in IPF patient lungs, increased levels of medium-chain and long-chain fatty acids and decreased levels of carnitine and acyl-carnitines were identified (Zhao et al., 2017). The authors proposed decreased β-oxidation in IPF lungs, because the transport of fatty acids to the mitochondrial matrix depends on the carnitine shuttle. Targeting regulators of fatty acid β-oxidation represents a promising strategy for new therapies for pulmonary fibrosis. Metformin, an activator of AMP-activated protein kinase α (AMPKα; also known as PRKAA) and an inhibitor of lipid synthesis, accelerated the resolution of bleomycin-induced lung fibrosis in mice (Rangarajan et al., 2018). In addition, two drugs that lower circulating lipids, fenofibrate and ciprofibrate, reduced lung fibrosis in mice, decreasing collagen accumulation and myofibroblast activation (Samah et al., 2012).

Sphingolipids have fundamental functions as signaling molecules involved in control of cell survival, inflammation and stress response (Ghidoni et al., 2015). Reduced levels of stearoyl-sphingomyelin, palmitoyl-sphingomyelin and sphingosine were found in IPF lungs compared to healthy control lungs (Zhao et al., 2017), which contrasts with our data indicating mostly an increase in sphingomyelins in young mice. A subclass of sphingolipids, ceramides, have pro-apoptotic properties and promote cell cycle arrest in epithelial and endothelial cells (Noe et al., 2009). Ceramides were found to be increased in patients with chronic obstructive pulmonary disease and IPF compared to the controls (Scarpa et al., 2013). Otherwise, detailed characterization of the lipidomic profile in IPF patient lungs is lacking. Our results corroborate an increase in ceramides in fibrotic lungs, including total dihydroceramide (DCER), hexosylceramide (HCER) and lactosylceramide (LCER) classes. Detected upregulation of phospholipids (PC and PE) and sphingolipids (sphingomyelins and sphingosines) in bleomycin-treated lungs might contribute to increased tissue remodeling, inflammation signaling and cell death. Increased phosphatidylserines (PS) could help fibrosis resolution by stimulating efferocytosis in a TAM (TYRO3, AXL and MERTK) receptor-dependent manner (Burstyn-Cohen and Maimon, 2019). On day 21 in the bleomycin model, this could reflect the initiation of the resolution phase or a counter-regulation against the fibrotic remodeling. Because phosphatidylglycerol (PG) lipids are major components of lung surfactant (Suryadevara et al., 2020), it seems also that there is an increased production or altered composition of surfactant in bleomycin-treated lungs (Romero et al., 2015). The latter would be in line with clinical observations, where IPF patients showed significant, adverse differences in surfactant tension compared to healthy individuals (Gunther et al., 1999). We also identified upregulation of myo-inositol in the bleomycin-treated lungs. This finding could be relevant for pulmonary fibrosis because inositol promotes maturation of surfactant phospholipids (PC and PI) (Menichini and Faccinetti, 2021), and decreases inflammatory processes and attenuates pulmonary edema after lung injury (Bizzarri et al., 2020). Thus, this is another metabolite of interest for further investigation in the context of IPF.

Besides changes in macromolecule degradation, collagen and ECM turnover, we also detected an increase in energy-producing pathways. We detected an increase in pyruvate and lactate, indicating a metabolic shift to aerobic glycolysis, which was previously observed in cancers and in the lungs of IPF patients (Kang et al., 2016; Kottmann et al., 2012; Xie et al., 2015; Zhao et al., 2017). Aerobic glycolysis enables faster ATP production compared to oxidative phosphorylation and generates pathway intermediates for cellular growth or proliferation. Pyruvate to lactate conversion was elevated in myofibroblasts of IPF patients, and the resulting increase in lactate led to increased pH-dependent TGFβ activation (Kottmann et al., 2012).

Intermediates of the TCA cycle were altered in our data in bleomycin-treated lungs, as was previously shown in TGFβ1-treated fibroblasts compared to control fibroblasts (Bernard et al., 2018) and in IPF lungs (Kang et al., 2016). That could be a consequence of increased input from glycolysis and fatty acid β-oxidation, reflecting increased energy demands of the fibrotic lungs. In addition, metabolites of the TCA cycle were also shown to stimulate activation of pro-fibrotic factors. For example, succinate acts by inhibiting hypoxia-inducible factor 1α (HIF1α) degradation to promote myofibroblast differentiation (Kottmann et al., 2012; Liu and Summer, 2019; Xie et al., 2015). Similarly, α-ketoglutarate activates mammalian target of rapamycin (mTOR)-regulated collagen gene transcription by inducing proline synthesis (Ge et al., 2018). In parallel, increased levels of TCA cycle metabolites and simultaneous decrease in glutamine levels indicate increased glutaminolysis (Bernard et al., 2018). Enhanced glutaminolysis supports cell growth and proliferation as well as myofibroblast activation by increased energy supply and biosynthesis through providing anabolic carbons (Bernard et al., 2018; DeBerardinis et al., 2007; Rodrigues et al., 2016). In a study by Zhao and co-workers, glutamate and glutamine were upregulated, but components of the TCA cycle were downregulated, with the exception of succinate and *cis*-aconitate (Zhao et al., 2017). In our study, both glutamate and glutamine were upregulated in bleomycin-treated lungs compared to control lungs, which, together with increased metabolites of the TCA cycle, indicates increased glutaminolysis.

Interestingly, we measured increased levels of itaconate in bleomycin-treated lungs compared to control lungs. Itaconate has been shown to act as an anti-fibrotic factor regulating the activation of fibroblasts (Ogger et al., 2020) and as an immunomodulatory molecule in macrophages of IPF patients (Lampropoulou et al., 2016; Meiser et al., 2016; Mills et al., 2018). In patients with IPF, the levels of itaconate in the airways were reduced, together with levels of *ACOD1*, the gene encoding its biosynthetic enzyme, in macrophages (Ogger et al., 2020). However, in bleomycin-induced lungs in the same study, *Acod1* levels were upregulated during inflammatory and fibrotic phases and downregulated during the later phases. The discrepancy between itaconate levels in mouse and human data could be attributed to the recovery potential of the bleomycin mouse model, where itaconate upregulation has counteractive effects on fibroblast proliferation. Furthermore, inhaled itaconate in mice treated with bleomycin at day 21 reduced collagen content and improved lung function, making it an interesting novel therapeutic opportunity (Ogger et al., 2020).

Eicosanoids, especially prostaglandins, are recognized as pro-inflammatory mediators in different disease context, and, thus, higher levels of polyunsaturated fatty acids arachidonate, docosadienoate, as well as prostaglandins E2, A2 and B2, in our study reflect increased inflammation and immune cell infiltration. Increased cytokine and chemokine levels in the BAL also argues for higher inflammation status in bleomycin-treated mice compared to controls. However, other studies proposed that lung eicosanoids might also act as anti-inflammatory and anti-fibrotic molecules (Huang and Peters-Golden, 2008). Inhalation of the liposomal particles with prostaglandin E2 protected mice against bleomycin-induced inflammation, weight loss, severity of fibrosis and mortality (Ivanova et al., 2013). The mechanisms of anti-fibrotic effects of prostaglandin E2 are based on the regulation of proliferation, apoptosis, plasminogen activation and inhibition of TGFβ-mediated myofibroblast activation (Suryadevara et al., 2020). Therefore, the observed increase could also indicate a mechanism to counteract increased inflammation and fibrosis in bleomycin-treated lungs.

Besides indicating increased protein degradation, ADMA also regulates cell survival and pro-inflammatory cytokine expression (Jiang et al., 2006, 2007). In a previous study, inhibition of dimethylaminohydrolase (DDAH), an enzyme that metabolizes ADMA, attenuated bleomycin-induced lung injury and collagen deposition, increased the number of proliferating cells and restored lung function (Pullamsetti et al., 2011). Thus, increased dimethylarginine in bleomycin-treated mice compared to controls could also represent a mechanism to combat increased inflammation in the lungs.

SAM upregulation in bleomycin-induced lungs is interesting, given its anti-oxidative properties and role in glutathione and polyamine synthesis (Yoon et al., 2016). Not much is known on the role of SAM in IPF, but in acute liver injury and fibrosis it was demonstrated that, through reducing oxidative stress and protecting mitochondrial function, it attenuated liver inflammation and fibrosis (Mato and Lu, 2007; Purohit et al., 2007). Moreover, in the mouse model of chronic asthma, SAM showed anti-inflammatory and anti-fibrotic effects, by inhibition of TGFβ1 signaling and reduction of oxidative stress (Yoon et al., 2016). Therefore, upregulation of SAM in the fibrotic lung could counteract the fibrotic injury upon bleomycin treatment.

In this study, we also aimed to investigate the effects of aging on the metabolic and lipidomic alterations in fibrotic lungs. It has been reported that the fibrotic changes and collagen accumulations in the lungs 14 days after bleomycin instillation were more severe in aged compared to young lungs (Cho et al., 2017; Redente et al., 2011). Here, we only detected one metabolite (cytidine) significantly deregulated in bleomycin-treated old compared to young mice. However, to obtain similar fibrotic responses in both age groups after the bleomycin treatment, we used a slightly lower dose of bleomycin that still elicited a fibrotic response comparable to that of young animals (0.63 mg/kg for old mice and 0.7 mg/kg for young mice). Thus, it seems that the susceptibility of older mice to develop more severe fibrosis was higher, but the overall metabolic and lipidomic response was similar to that in young mice. Minor statistically significant differences between young and old mice in our study could also mean that aging has more pronounced effects on other processes or stages of disease development that are not reflected in metabolism. In line with this, resolution of accumulated collagen has been delayed in old mice, because collagen content returned to baseline levels in young mice 8 weeks after bleomycin treatment, whereas in old mice at the same time point it was still increased compared to that in saline-treated controls (Podolsky et al., 2020). We found several differential responses between the age groups according to the distribution of fold changes for different metabolic pathways and lipid classes. Hence, although the general trend in metabolic alterations is similar in both age groups, subtle differences in the intensity of the changes do exist.

In summary, we detected increases in protein, nucleic acid and lipid degradation, together with increased collagen synthesis and ECM turnover, in bleomycin-treated compared to control mouse lungs as a model of IPF. Energy-producing pathways were upregulated in the fibrotic lungs, together with fatty acid oxidation and inflammatory eicosanoids. We found many metabolites that have been described in the context of human IPF lungs and mouse models of pulmonary fibrosis, validating the model system and our analysis. In addition, we identified potential novel players in the pathogenesis of pulmonary fibrosis. Therefore, the metabolomic and lipidomic characterization performed in this study represents a comprehensive map of the fibrotic changes and tissue remodeling in the mouse lung after bleomycin challenge. Thus, this work will aid future investigations of metabolism-specific therapies in the field of fibrotic lung diseases.

## MATERIALS AND METHODS

### Animals

Mice experiments were planned and conducted to conform to the animal welfare laws, guidelines and policies by the local authority Regierungspräsidium Tübingen, Germany, licence number TVV 17-023-G. The *in vivo* studies were conducted in the AAALAC-certified facilities at Boehringer Ingelheim Pharma. Male C57BL/6J mice (*Mus musculus*) aged 8-12 weeks and 21 months were purchased from Janvier (Le Genest-Saint-Isle, France). Mice were housed separately in individually ventilated cages at 22-25°C, 46-65% humidity and 12-h day/night cycle. Animals received water and food *ad libitum*.

Male mice were randomized before the study. Mice were instilled intratracheally under 3-4% isoflurane anesthesia once on day 0 with either bleomycin sulphate or saline solution (NaCl). Bleomycin administration was performed in a hanging position with a Vasofix-Braunüle 22G (B. Braun, Melsungen, Germany). Young and old mice were instilled with 0.7 mg/kg and 0.63 mg/kg bleomycin sulphate (Merck, Darmstadt, Germany) in saline solution, respectively. Control mice received saline solution only.

### Lung homogenization and BAL extraction

Lung tissue was perfused twice with 10 ml 1× PBS (Sigma-Aldrich, St Louis, MO, USA) through the right side of the heart entering the pulmonary vasculature. To extract the BAL, lungs were flushed two times with 0.8 ml HBSS buffer containing protease inhibitor. The BAL was centrifuged, and supernatants were stored until usage, at −80°C. Subsequently, a lung was removed from the chest cavity, and each lobe was separated and thereafter immediately flash frozen in liquid nitrogen. Lungs were then stored at −80°C until homogenized in 2 ml Eppendorf tubes (Eppendorf, Hamburg, Germany) using a TissueLyser LT (Qiagen, Hilden, Germany). Samples remained frozen throughout the entire procedure.

### Cytokine/chemokine measurement

Supernatants of the BAL were tested for ten cytokines/chemokines using an individual U-PLEX assay, following the manufacturer’s instructions, from Meso Scale Discovery (Rockville, MD, USA).

### Metabolomics and lipidomics

Around 50 mg homogenous tissue powder per sample was processed by Metabolon Inc. (Morrisville, NC, USA) for metabolomic analysis, and 8 mg was extracted for lipidomic analysis. The metabolomics method was described in more detail in the supplemental material of Rhee and coworkers (Rhee et al., 2019). In brief, samples were supplemented with several recovery standards and extracted with methanol under vigorous shaking for 2 min. Samples were then centrifuged and divided into fractions for analysis by four ultra-performance liquid chromatography (UPLC)-tandem mass spectrometry (MS/MS) methods (Metabolon HD4 platform). Samples were dried in a speed vac, stored under nitrogen, and reconstituted in solvents compatible to each method containing standards to ensure injection and chromatographic consistency. All liquid chromatography (LC)-mass spectrometry (MS) methods were run on a Waters ACQUITY UPLC system connected to a Thermo Fisher Scientific Q-Exactive mass spectrometer with a heated electrospray ionization (HESI-II) source. The chromatographic methods were optimized as follows: the acidic positive ion mode was optimized for more hydrophilic compounds. Therefore, reconstituted extracts were gradient eluted from a C18 column (Waters UPLC BEH C18-2.1×100 mm, 1.7 µm) using water and methanol, containing 0.05% perfluoropentanoic acid (PFPA) and 0.1% formic acid (FA). The acidic positive ion conditions were optimized for more hydrophobic compounds. Here, metabolites were gradient eluted from the same C18 column using methanol, acetonitrile, water, 0.05% PFPA and 0.01% FA. The basic negative ion-optimized conditions used a dedicated C18 column, and extracts were gradient eluted using methanol and water, with 6.5 mM ammonium bicarbonate at pH 8. The negative ionization HILIC conditions (Waters UPLC BEH Amide 2.1×150 mm, 1.7 µm) used a gradient consisting of water and acetonitrile with 10 mM ammonium formate, pH 10.8. The Orbitrap mass analyzer was operated at 35,000 mass resolution alternating between MS and data-dependent MS^n^ scans using dynamic exclusion. The scan range varies slightly between methods and covered ∼70-1000 m/z. Raw data were extracted, peak identified and QC processed using Metabolon's hardware and software. Compounds were identified by comparison to more than 4500 library entries of purified standards or recurrent unknown entities using the retention time/index (RI), mass to charge ratio (m/z, +/− 10 ppm) and chromatographic data (including MS/MS spectral data). Library matches for each compound were checked manually for each sample and corrected if necessary. Peaks were quantified as area under the curve.

Lipidomic analysis was performed using a global targeted lipidomics method termed the Lipidyzer platform (Ubhi, 2018) (Metabolon TrueMass^®^ Complex Lipid Panel). Lipids were extracted by first weighing the samples and soaking in 1:1 dichloromethane:methanol overnight at 4°C. The supernatants were subjected to a modified Bligh-Dyer extraction using methanol/water/dichloromethane in the presence of 54 deuterated internal standards covering ten lipid classes. The organic phase was dried under nitrogen and subsequently reconstituted in 0.25 ml of 10 mM ammonium acetate dichloromethane:methanol (50:50). Samples were transferred in vials and analyzed via a liquid chromatograph (Shimadzu, Kyoto, Japan) with nano PEEK tubing and Sciex SelexION-5500 QTRAP (AB Sciex LLC, Framingham, MA, USA). The Lipidyzer platform utilizes positive and negative flow injection analysis (FIA) combined with the Sciex SelexION differential mobility separation (DMS) and multiple reaction monitoring (MRM) to analyze in total more than 1100 individual lipid species. Positive and negative electrospray ionization modes were combined with DMS or not to analyze lipid classes in the following combination: combination 1, DMS in negative ionization mode: PC, PE, lysophosphatidylcholine (LPC), lysophosphatidylethanolamine (LPE); combination 2, DMS in positive ionization mode: sphingomyelin (SM); combination 3, without DMS in negative ionization mode: free fatty acid (FFA); and combination 4, without DMS in positive ionization mode: monoacylglycerol (MAG), TAG, DAG, cholesteryl ester (CE), ceramide (CER). Formed adducts used for quantification were [M+NH4]+ for CE, DAG and TAG, [M+H]+ for SM, CER and MAG, [M−H]− for LPE, PE and FFA, and [M+CH3COO]− for LPC and PC. Individual lipid species were quantified by calculating the ratios of the peak areas of the target lipid and its assigned internal standard, subsequently multiplying by the amount of the internal standard in the sample per sample weight. Lipid species concentrations were background subtracted using the concentrations detected in water blank extracts. For the present paper, we focused on lipid species concentrations (*n*=1011) and lipid class concentrations (*n*=15), resulting in overall 1026 features for analyses (Table S2) that were transformed for analysis as described below.

### Lung function analysis

Lung function (resistance and FEV, 0.1) was measured using a Flexivent FX1 system (Scireq, Montreal, Quebec) in mice anesthetized with Narcoren (Boehringer Ingelheim Pharma, Ingelheim, Germany). Each measurement was performed four times per animal and the average value taken. During those tests, mice were ventilated with a tidal volume of 10 ml/kg at a frequency of 150 breaths/min. One-way ANOVA was used to test for the differences between groups, and *P*-values were calculated using Tukey's multiple comparison test. The graphs were plotted using GraphPad Prism software (v.9.0.0).

### Quantitative RT-PCR analysis

For each sample, 20 mg lung tissue was used for total RNA isolation. RNA was extracted using a Qiazol kit according to the manufacturer's protocol (Qiagen, Hilden, Germany). Quality of RNA was assessed by a Fragment Analyzer (Agilent Technologies, Santa Clara, CA, USA) using an SS RNA Kit (Agilent Technologies). cDNA was synthesized from 1 mg RNA per sample using a high-capacity cDNA reverse transcription kit (Thermo Fisher Scientific, Munich, Germany) according to the manufacturer's protocol. Quantitative RT-PCR was performed using the standard protocol of the TaqMan™ Fast Advanced Master Mix (Applied Biosystems, Foster City, USA) on a Viia7 Real-time PCR system (Applied Biosystems). Relative expression of target genes for each group was calculated using the ΔCt method. Mouse hypoxanthine-guanine phosphoribosyltransferase (*Hprt*) was used as a reference gene. One-way ANOVA was used to test for the differences between groups, and *P*-values were calculated using Tukey's multiple comparison test. The graphs were plotted using GraphPad Prism software (v.9.0.0). TaqMan™ probes used in quantitative RT-PCR analysis are listed in Table S7.

### Statistics and reproducibility

For metabolomic and lipidomic analysis, the sample size was *n*=5 for control animals and *n*=9 for bleomycin-treated animals, for each age group. Allocation of animals to experimental groups was randomized. No sample was excluded for the analysis. The code that reproduces the analysis performed in this study is publicly available at https://www.github.com/bi-compbio/bleo_d21_metab_lipid. The statistical modelling was performed with the R language for statistical computing (https://www.r-project.org/). All the functions mentioned in the data analysis section belong to the stats package unless otherwise specified. For quantitative RT-PCR analysis, the sample size was *n*=5 for young control, *n*=6 for old control and *n*=9 for young and old bleomycin-treated animals, and the measurement was also run in technical triplicates.

### Data analysis

#### Data transformation

The results of metabolomic and lipidomic data are available in Tables S1 and S2. The abundances of 647 metabolites for the 28 samples were taken as normalized values in terms of raw area counts. After discarding metabolites with more than 50% missing values, 637 metabolites remained (Table S1). Missing values were imputed by the minimum intensity of the metabolite. Metabolic features were log transformed by *y*^′^=log _2_(*y*+1) for downstream analysis.

The readouts of 1026 lipids (lipid species concentrations; Table S2) were taken as scaled imputed values. Each lipid abundance was rescaled to have a median value of 1 per lipid, and then missing values were imputed by the minimum of that lipid. Lipid features were log-transformed by *y*^′^=log _2_(*y*) for downstream analysis.

#### PCA

Two-dimensional representations were plotted using PCA using pca() from the pcaMethods package (Stacklies et al., 2007). PCA was applied to the log-transformed features, later centered and scaled, respectively, on the 647 metabolites and 1026 lipids.

#### Heat maps and box plots

Centered and scaled analyte features (so that each analyte had a mean of 0 and a variance of 1) were graphically displayed using heat maps and box plots. When applicable, rows or columns were hierarchically clustered within heat maps using Euclidean distances and the ‘complete’ method in hclust() (Hastie et al., 2009).

#### Differential abundance

Differential abundances were assessed using linear models, fitted on each analyte (metabolite, lipid) separately for the old and young mice, using the lm() function:

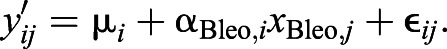

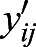
 is the feature value of the *i*-th analyte in the *j*-th sample. μ_*i*_ is the mean value of the *i*-th analyte in the reference group (NaCl). α_Bleo,*i*_ is the difference of mean values between the bleomycin-treated and NaCl groups. The indicator variable *x*_Bleo,*j*_ was set to 1 if sample *j* was treated with bleomycin, or to 0 otherwise. ε_*ij*_ is the error term.

Log fold changes were computed for bleomycin treatment as logFC_i_=α_Bleo,*i*_. *P*-values were obtained through a one-way ANOVA (Chambers and Hastie, 1992) using the anova() function and corrected for multiple testing, stratifying by the -omic and the age group, using the FDR (Benjamini et al., 2001) with p.adjust(method=“fdr”). The *i*-th analyte was called differentially abundant when FDR*_i_*<5%, with no threshold on its logFC*_i_*.

The log fold changes of old and young mice (stratified by the -omic) were compared using linear (Pearson's) correlation; its magnitude and *P*-value were calculated with the cor.test(method=“pearson”) function. Their graphical comparison included the least squares fit of young log fold changes as a function of old log fold changes, with its equation (slope, intercept) and coefficient of determination *R*^2^. After calling differential analytes, the significance of the overlap between old and young mice differential entities was assessed with Fisher's exact test, estimating the odds ratio and its two-sided *P*-value with the function fisher.test().

Analogously, differential abundances in old versus young mice were calculated, fitting separate models for the NaCl and bleomycin groups:

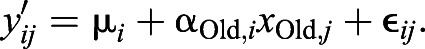
Here, μ_*i*_ is the mean value of the *i*-th analyte in young mice, α_Old,*i*_ is the difference of mean values between old and young mice, and *x*_Old,*j*_ was 1 if sample *j* came from an old mouse, or 0 otherwise.

#### Functional analysis

We used two libraries of metabolite and lipid sets for functional annotation of the differential analytes. First, pathway annotations for *Mus musculus* were retrieved from the KEGG (Kanehisa et al., 2019), release 91.0+/07-15 (July 2019), with KEGG compound (metabolites and lipids), KEGG pathways and KEGG brite. Second, dedicated annotation libraries with super- and subpathways for metabolites and classes and subclasses for lipids were extracted from the quantification tables provided by Metabolon Inc.

#### Over-representation analysis

Pathway over-representation analysis *P*-values were computed using Fisher's exact test as implemented in the enricher() clusterProfiler function (Yu et al., 2012). The statistical background was set to all the analytes measured within every -omic, while positives were differentially abundant analytes. Pathway *P*-values were corrected for multiple testing using the FDR, stratifying by pathway library and -omic. Pathways were considered significant if FDR<5%.

#### GSEA

We applied the GSEA algorithm as implemented in clusterProfiler (Yu et al., 2012) to the KEGG pathways for metabolites. Pre-ranked GSEA was run on the list of metabolites, sorted by their signed log fold changes. We used the following parameters: GSEA(exponent=1, nPerm=100,000, minGSSize=10, maxGSSize=500, pvalueCutoff=1, pAdjustMethod=“fdr”, seed=TRUE, by=“fgsea”).

#### Differential pathway response

We investigated whether young and old mice responded differently to bleomycin treatment within each pathway. Within-pathway differences were assessed by a Wilcoxon signed-rank test (Wilcoxon, 1992) on the paired differences between old and young bleomycin-treated signatures from any -omic belonging to the pathway, logFC_i_(old)−logFC_i_(young). The wilcox.test (paired=TRUE) function was used, and *P*-values were corrected using the FDR, stratifying by pathway library. The same analysis was carried out separately for metabolomic and lipidomic data using the Metabolon annotations.

#### Network analysis

We contextualized the list of deregulated metabolites in both old and young mice using the network-based approach FELLA (Picart-Armada et al., 2018). The knowledge graph was built from the KEGG *Mus musculus* release 96.0+/12-08, using buildDataFromGraph(normality=“diffusion”, niter=1000). Only metabolic pathways were allowed, i.e. those for which the top-level KEGG brite annotation was ‘Metabolism’. We ran the propagation with enrich(methods=“diffusion”, approx=“normality”). The resulting subnetwork came from generateResultsGraph() with default parameters.

## Supplementary Material

Supplementary information
