# Supplementary material for: Mapping the metabolomic and lipidomic changes in the bleomycin model of pulmonary fibrosis in young and aged mice
Source: Dis Model Mech. 2022 Jan 25;15(1):dmm049105. doi: 10.1242/dmm.049105 (PMC8807555; doi:10.1242/dmm.049105)
Supplement: Supplementary information [file dmm-15-049105-s1.pdf]

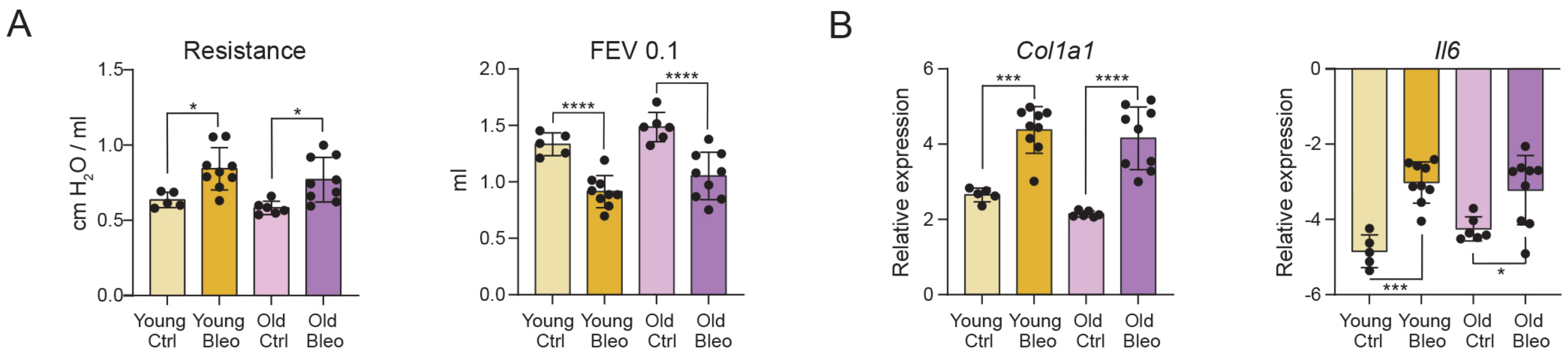

Fig. S1. Fibrotic response of young and old lungs is comparable

(A) Lung function parameters resistance and forced expiratory volume (FEV 0.1) show similar responses in young and old Bleomycin-treated lungs. (B) Relative mRNA expression of *Col1a1* and *Il6* obtained by qRT-PCR analysis. *Hprt1* was used as a reference gene. Data shown in A and B are mean values +/- standard deviation (s.d.). p-values are calculated using Tukey's multiple comparison test. * $p<0.05$; ** $p<0.01$; *** $p<0.001$; **** $p<0.0001$.

Disease Models & Mechanisms • Supplementary information

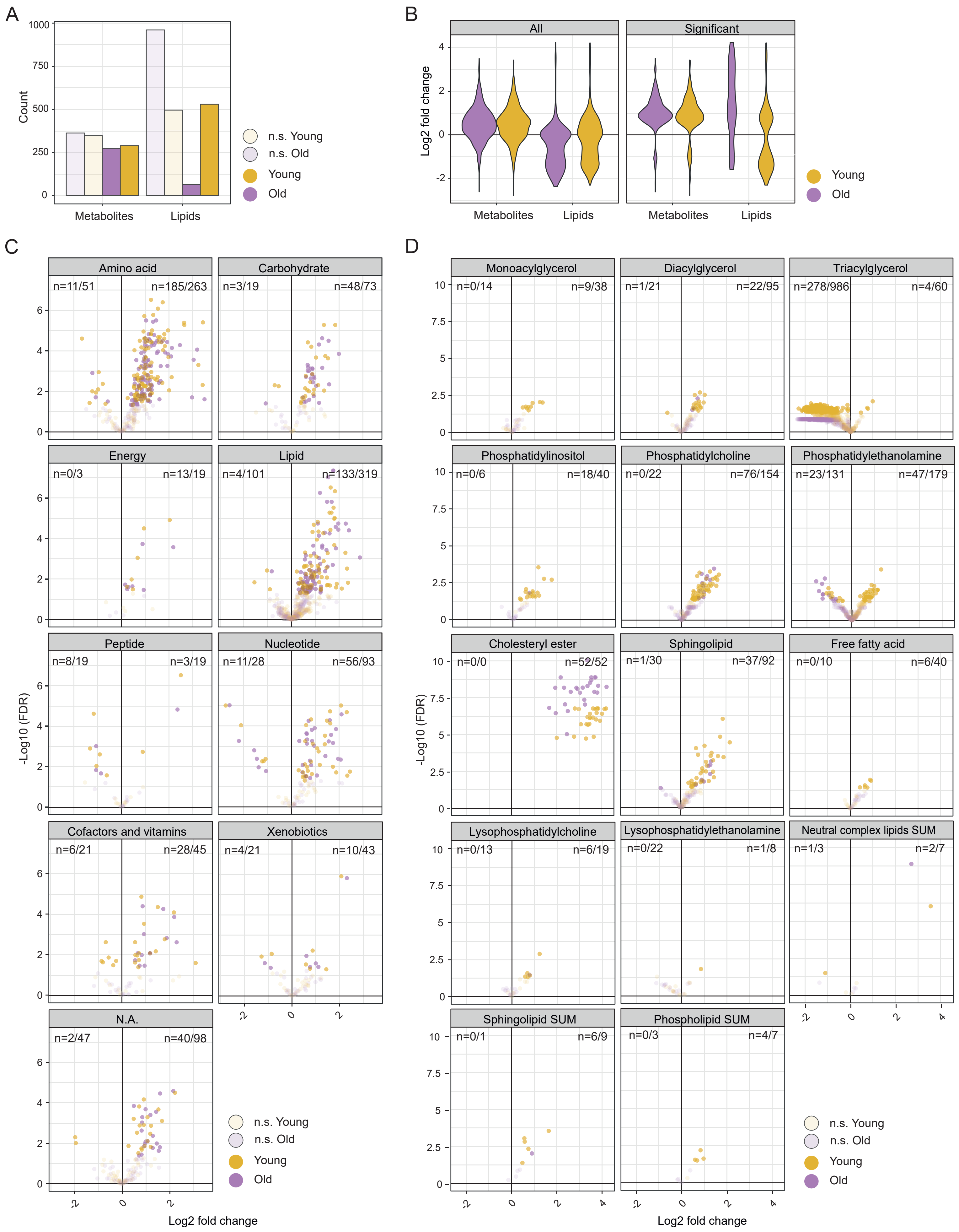

Disease Models & Mechanisms • Supplementary information

Fig. S2. Significantly deregulated metabolites in Bleomycin-treated lungs compared to controls
(A) Counts of detected not significant and significant metabolites and lipids of Bleomycin-treated lungs compared to controls in young and old lungs. (B) Violin plots indicating the distribution of log2 fold changes of all detected metabolites and lipids (left) and significantly deregulated metabolites and lipids (right) in Bleomycin-treated lungs compared to controls. (C,D) Volcano plots of indicated metabolic superpathways (C) and lipid classes (D) with numbers of significantly downregulated and upregulated entities of Bleomycin-challenged lungs compared to controls. FDR < 0.05 in A-D. FDR, false discovery rate; n.s., not significant; NA, not annotated; SUM, total. Neutral complex lipid SUM = Total Cholesteryl ester, Monoacylglycerol, Diacylglycerol, Triacylglycerol, Free fatty acid; Sphingolipid SUM = Total Sphingomyelin, Ceramide, Dihydroceramide, Lactosylceramide, Hexosylceramide; Phospholipid SUM = Total Phosphatidylcholine, Lysophosphatidylcholine, Phosphatidylethanolamine, Lysophosphatidylethanoamine, Phosphatidylinositol.

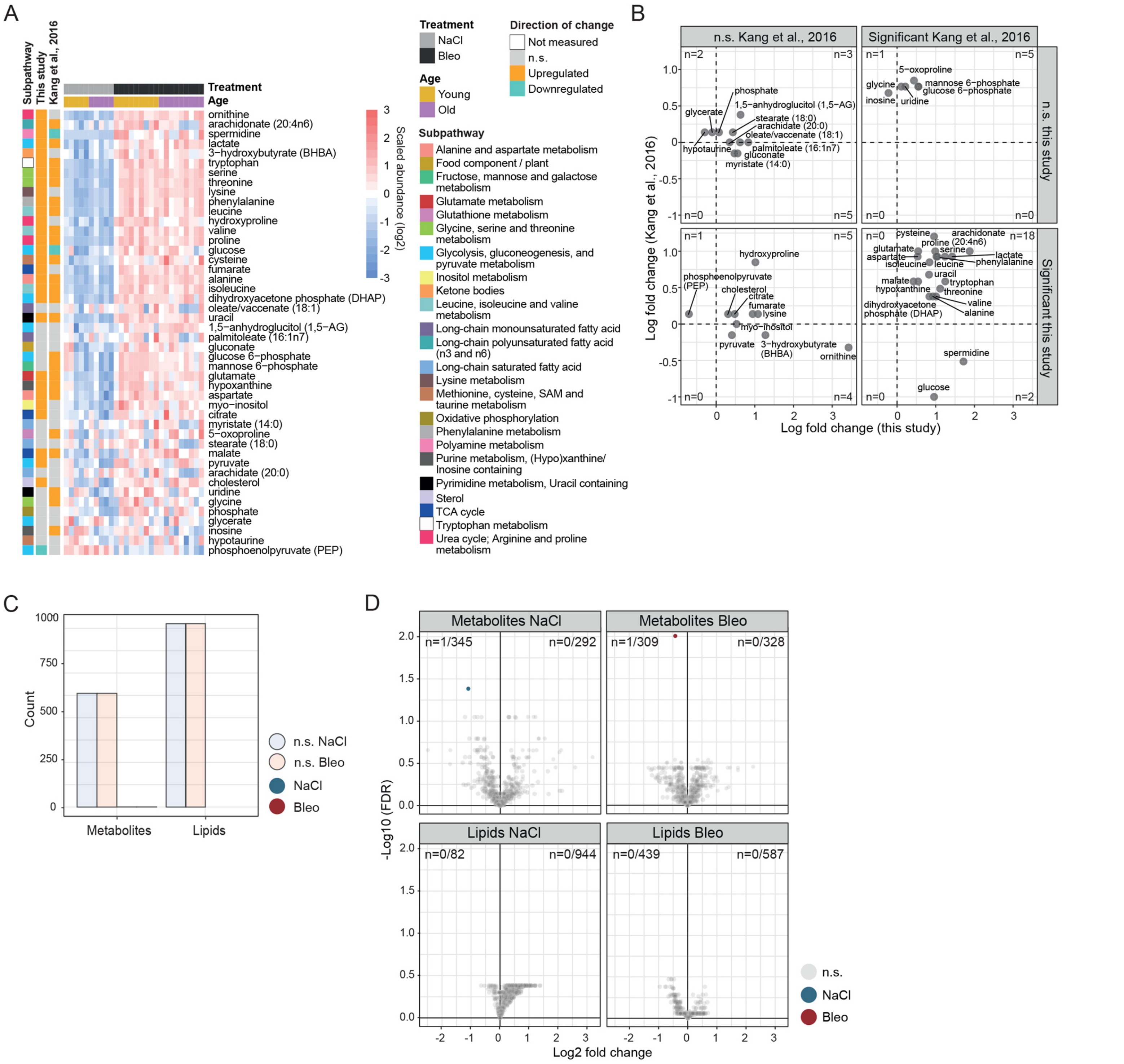

Fig. S3. Comparison of Bleomycin model to IPF lungs and analysis of significantly deregulated metabolites in old Bleomycin-treated lungs compared to young

(A) Heat map of all detected metabolites in the lungs of IPF patients compared to controls (Kang et al., 2016) that were identified in our Bleomycin mouse model and indicated directions of changes. (B) Comparison of log2 fold changes in the published data from IPF human lungs (Kang et al., 2016) and the Bleomycin-treated mouse lungs (this study) compared to controls. Numbers for detected metabolites in each quadrant are indicated. (C) Counts of detected not significant and significant metabolites and lipids of Bleomycin-treated old lungs compared to young. (D) Volcano plots of all detected metabolites and lipids when comparing old to young Bleomycin-treated lungs. Significant entities are colored (one-way Anova) and the numbers for upregulated and downregulated metabolites and lipids are indicated. The lists are provided in Table S5. FDR < 0.05. Bleo, Bleomycin; FDR, false discovery rate; n.s., not significant; SAM, S-adenosylmethionine; TCA, tricarboxylic acid cycle.

Disease Models & Mechanisms • Supplementary information

A

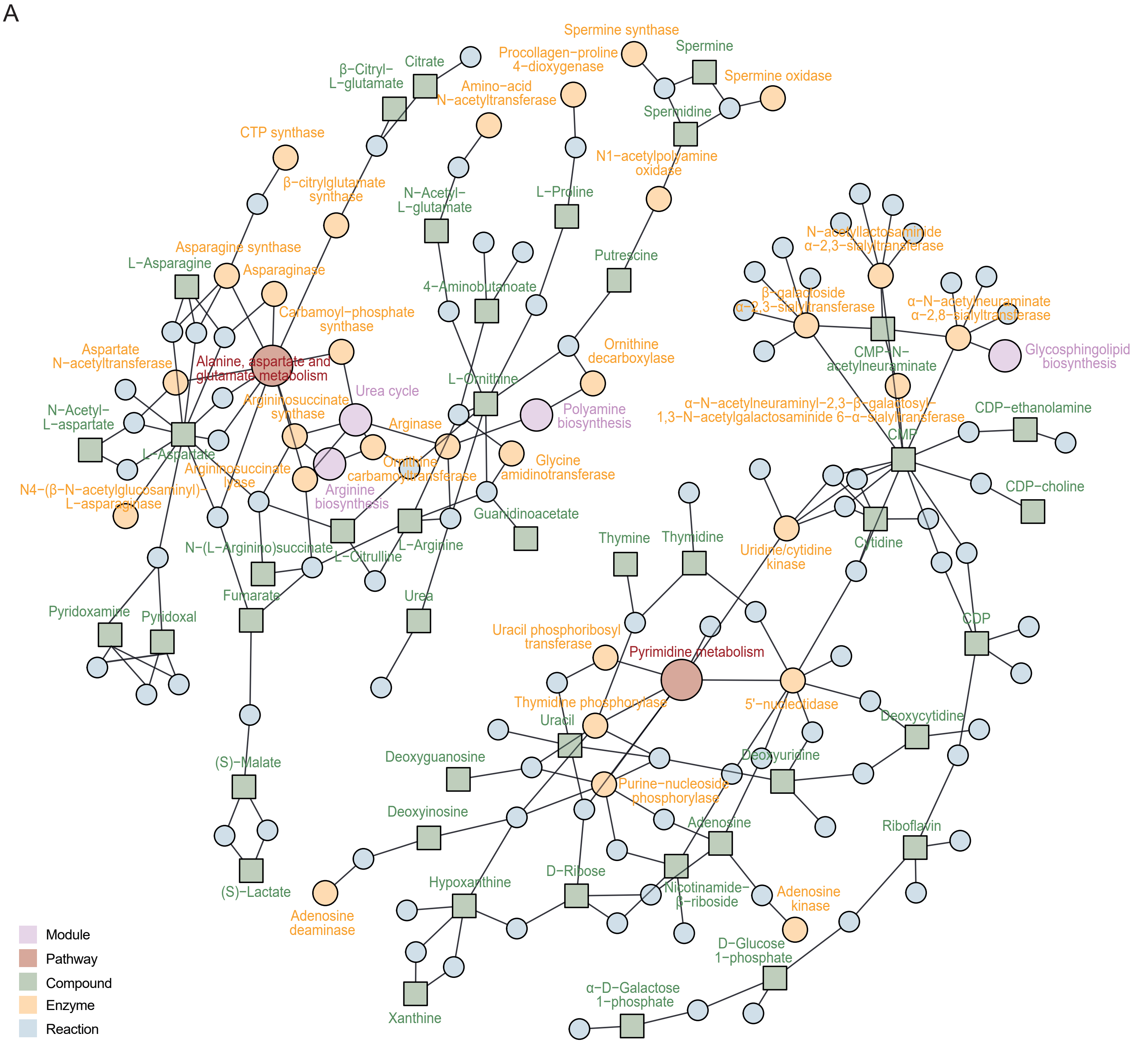

Fig. S4. Enriched metabolomic pathways in Bleomycin-treated lungs compared to controls in young and old lungs

(A) Intersected FELLA analysis of enriched metabolic pathways in young and old mice in Bleomycin-treated lungs compared to controls. Pathways, modules, enzymes, reactions and compounds that were proximal to the deregulated metabolites within the KEGG knowledge graph are indicated. Connecting lines represent links between KEGG entities, as found in the KEGG database annotations. CMP, Cytidine monophosphate; CDP, Cytidine diphosphate; CTP, Cytidine triphosphate.

Disease Models & Mechanisms • Supplementary information

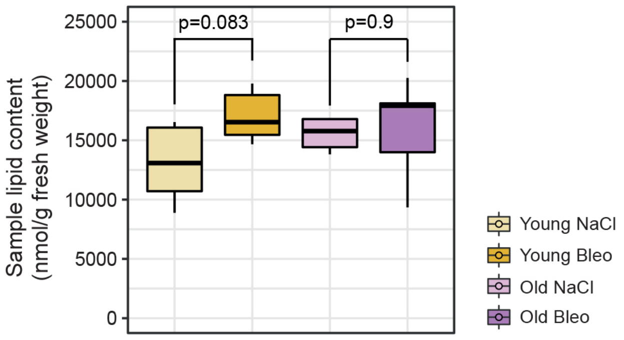

Fig. S5. Total lipid abundances in all experimental groups.
Total lipid levels in all four experimental groups as calculated by the sum of all analyzed lipids. P-values were calculated by the non-parametric two-sided Wilcoxon rank-sum test, stratifying by age.

Table S1. Metabolomic data

Click here to download Table S1

Table S2. Lipidomic data

Click here to download Table S2

Table S3. Differential metabolites and lipids in Bleomycin-treated compared to control lungs

Click here to download Table S3

Table S4. List of metabolites and lipids that are significantly deregulated only in young, old or in both age groups in Bleomycin-treated lungs compared to controls

Click here to download Table S4

Table S5. Differential metabolites and lipids in Bleomycin-treated old compared to young lungs

Click here to download Table S5

Table S6. Overrepresentation analysis, GSEA and differential fold change pathways responses in young and old Bleomycin-treated lungs compared to controls

Click here to download Table S6

Table S7. TaqMan probes used in quantitative RT-PCR analysis

Gene	Probe	Catalog number	Label
Col1a1	Mm00801666_g1	4351368	FAM
Il6	Mm00446190_m1	4448491	VIC
Hprt1	Mm03024075_m1	4448491	VIC

Disease Models & Mechanisms • Supplementary information
